# The deubiquitinase TRABID stabilizes the K29/K48-specific E3 ubiquitin ligase HECTD1

**DOI:** 10.1074/jbc.RA120.015162

**Published:** 2021-01-08

**Authors:** Lee D. Harris, Janic Le Pen, Nico Scholz, Juliusz Mieszczanek, Natalie Vaughan, Simon Davis, Georgina Berridge, Benedikt M. Kessler, Mariann Bienz, Julien D.F. Licchesi

**Affiliations:** 1 Department of Biology and Biochemistry, University of Bath, Bath, United Kingdom; 2 MRC Laboratory of Molecular Biology, Cambridge Biomedical Campus, Cambridge, United Kingdom; 3 Target Discovery Institute, Nuffield Department of Medicine, University of Oxford, Oxford, United Kingdom

**Keywords:** ubiquitin, deubiquitination, polyubiquitin chain, E3 ubiquitin ligase, ubiquitin thioesterase, protein degradation, HECTD1, TRABID, K29/K48-linked polyubiquitin chain, DUB, deubiquitinase, E1, E1-activating enzyme, E2, E2-conjugating enzyme, E3, E3 ubiquitin ligase, HECT, homologous to the E6-AP carboxyl terminus (HECT) domain family, NZF, Np14 zinc finger, OTU, ovarian tumor, Ub-AQUA, ubiquitin-absolute QUAntification, UBD, ubiquitin binding domain, UbiCREST, ubiquitin chain restriction analysis

## Abstract

Ubiquitin is a versatile posttranslational modification, which is covalently attached to protein targets either as a single moiety or as a ubiquitin chain. In contrast to K48 and K63-linked chains, which have been extensively studied, the regulation and function of most atypical ubiquitin chains are only starting to emerge. The deubiquitinase TRABID/ZRANB1 is tuned for the recognition and cleavage of K29 and K33-linked chains. Yet, substrates of TRABID and the cellular functions of these atypical ubiquitin signals remain unclear. We determined the interactome of two TRABID constructs rendered catalytic dead either through a point mutation in the catalytic cysteine residue or through removal of the OTU catalytic domain. We identified 50 proteins trapped by both constructs and which therefore represent candidate substrates of TRABID. The E3 ubiquitin ligase HECTD1 was then validated as a substrate of TRABID and used UbiCREST and Ub-AQUA proteomics to show that HECTD1 preferentially assembles K29- and K48-linked ubiquitin chains. Further *in vitro* autoubiquitination assays using ubiquitin mutants established that while HECTD1 can assemble short homotypic K29 and K48-linked chains, it requires branching at K29/K48 in order to achieve its full ubiquitin ligase activity. We next used transient knockdown and genetic knockout of *TRABID* in mammalian cells in order to determine the functional relationship between TRABID and HECTD1. This revealed that upon *TRABID* depletion, HECTD1 is readily degraded. Thus, this study identifies HECTD1 as a mammalian E3 ligase that assembles branched K29/K48 chains and also establishes TRABID-HECTD1 as a DUB/E3 pair regulating K29 linkages.

Ubiquitin is a small and highly conserved protein modifier, which has emerged as a complex yet specific posttranslational modification regulating protein fate and function. The role of ubiquitin is central to proteostasis by serving as the key signal for the degradation of proteins or organelles whether it be through the ubiquitin proteasome system (UPS) or autophagy (1, 2, 3). Ubiquitin is added to lysine residues on protein targets through the sequential activity of E1-activating enzymes, E2-conjugating enzymes, and E3 ubiquitin ligases, as a single moiety or as a ubiquitin chain (4, 5). These distinct signals mediate specific and diverse downstream cellular processes. For example, while addition of a single ubiquitin molecule on a protein substrate can regulate its trafficking, polyubiquitination regulates a plethora of cellular processes including cell cycle progression, DNA repair, and inflammation (6, 7, 8, 9). Polyubiquitin chains are assembled through an isopeptide bond between the C terminus of Gly76 of a donor ubiquitin and the α-amino group of any of the seven lysines (K6, K11, K27, K29, K33, K48, and K63) or the N-terminus (*i.e.*, linear ubiquitin, Met-1) of an acceptor ubiquitin. Protein ubiquitination also occurs on noncanonical attachment sites within substrates, including on serine, threonine, and cysteine residues (10, 11, 12).

Some deubiquitinases (DUB), in particular those from the ovarian tumor (OTU) family, and E3 ubiquitin ligases such as members of the homologous to the E6-AP carboxyl terminus (HECT) family have shown specificity for particular ubiquitin chains. This, combined with the difference in the three-dimensional structure of these ubiquitin polymers, further suggests that they represent distinct signals (13). All ubiquitin linkage types have been identified in yeast (14) and mammalian cells (15). While homotypic K48-linked polyubiquitin chains target proteins for degradation by the UPS, cargoes modified with K63-linked chains are recognized as part of the autophagic response (16, 17, 18). Nevertheless, the role and function of polyubiquitin chains beyond those assembled through homotypic (*i.e.*, assembled through one lysine only) such as K48 and K63 linkages are only starting to emerge (19, 20, 21). More recently, polyubiquitin has been found to exist as heterotypic and also branched chains. For example, branched K11/K48-linked chains increase the degradation rate of mitotic cyclins by the UPS, while branched K48/K63 chains regulate NF-kB gene activation through recognition by TAB2, a subunit of the TAK1 complex (22, 23).

TRABID (also known as ZRANB1) belongs to OTU DUB family (24). It contains three highly conserved Npl14 zinc finger domains (3xNZF), which function as ubiquitin binding domains (UBD), and the OTU catalytic domain responsible for the hydrolysis of ubiquitin polymers (24, 25, 26). The expansion of the ubiquitin toolbox through, for example, the chemical synthesis of ubiquitin dimers, has been instrumental in determining TRABID’s specificity for K29-linked ubiquitin chains (27). Further analysis using the full set of eight ubiquitin dimers revealed that in addition to K29-linked ubiquitin dimers, TRABID also cleaves K33-linked ubiquitin chains and that the activity toward K29 and K33-linked dimers is greater than for K63-linked diubiquitin (28). The discovery of the AnkUBD as a novel UBD abutting the N-terminus of TRABID OTU domain revealed that it is required for full DUB activity and to some extent specificity too. These findings as well as recent studies, which identified TRABID’s NZF 1 as the minimal UBD required for the recognition of K29- and K33-linked diubiquitin, establish TRABID as a unique DUB highly tuned for the recognition and processing of these atypical ubiquitin chains (29, 30, 31).

TRABID has been proposed to regulate the Wnt/**β**-catenin/Tcf signaling pathway through APC (Adenomatous Polyposis Coli), the epithelial to mesenchymal transition (EMT) through Twist, as well as the innate immune response, with these studies implicating the deubiquitination of K63-linked chains as potential mechanism (32, 33, 34, 35). More recently, TRABID was shown to process K29 and K33-linked chains on UVRAG, a Beclin 1 complex component, thereby inhibiting autophagy and increasing hepatocellular cellular carcinoma growth (36). Another interesting finding came through phenotypic studies of *Trabid* KO mice, which revealed that in dendrocytes, *Trabid* loss of function led to proteasomal degradation of the histone demethylase Jmjd2 (37). This in turn decreased expression of proinflammatory cytokines interleukin 12 and 23 and dampened inflammatory T-cell responses. The histone methyltransferase EZH2 also appears to be a target of TRABID DUB activity, and TRABID depletion has been shown to decrease EZH2 levels (38). These studies highlight a role for TRABID in transcriptional regulation as well as novel cross talk between protein ubiquitination and epigenetics mechanisms although the types and composition of ubiquitin chains involved remain to be determined.

Proteomics studies have been useful in identifying TRABID candidate interactors including components of the striatin-interacting phosphatase and inase (STRIPAK) complex and the E3 ubiquitin ligases HECTD1 and HERC2 (33, 39). Yet whether these proteins represent substrates of TRABID DUB activity rather than interactors has remained unclear. In this study, we aimed to further expand on our understanding of TRABID as well as the atypical ubiquitin chains that it regulates. We first used two catalytic dead TRABID constructs, a single point mutation in the catalytic OTU domain (TRABID^C443S^) and a construct lacking the OTU domain entirely (TRABID ΔOTU), to specifically trap ubiquitinated substrates. By comparing the interactome of these two constructs, we identified NZF- and OTU-specific TRABID interactors, including several E3 ubiquitin ligases as candidate interactors and potential substrates of TRABID DUB activity. We validated HECTD1 as a substrate of TRABID activity and used *in vitro* autoubiquitination assays, ubiquitin chain restriction analysis (UbiCREST) and ubiquitin-absolute QUAntification (Ub-AQUA) to show that the catalytic HECT domain of HECTD1 preferentially assembles K29- and K48-linked ubiquitin chains. Interestingly, our data indicate that although UBE3C and HECTD1 both use K29 and K48 linkages to assemble ubiquitin chains, the topology of the chains is different. Indeed, we found that to achieve its full activity, HECTD1 assembles ubiquitin chains, which contain branched K29/K48 linkages. Finally, we explored the functional relationship between TRABID and HECTD1 using transient siRNA knockdown as well as CRISPR/Cas9 *TRABID* KO in mammalians cells and mice. Loss-of-function and rescue assays showed that TRABID stabilizes HECTD1 protein levels, further establishing this novel and functional DUB-E3 pair as key regulators of K29-linked polyubiquitination.

## Results

### Interactome studies of two catalytic dead TRABID constructs differentiate between OTU-specific interactors and candidate substrates

**Figure 1:**
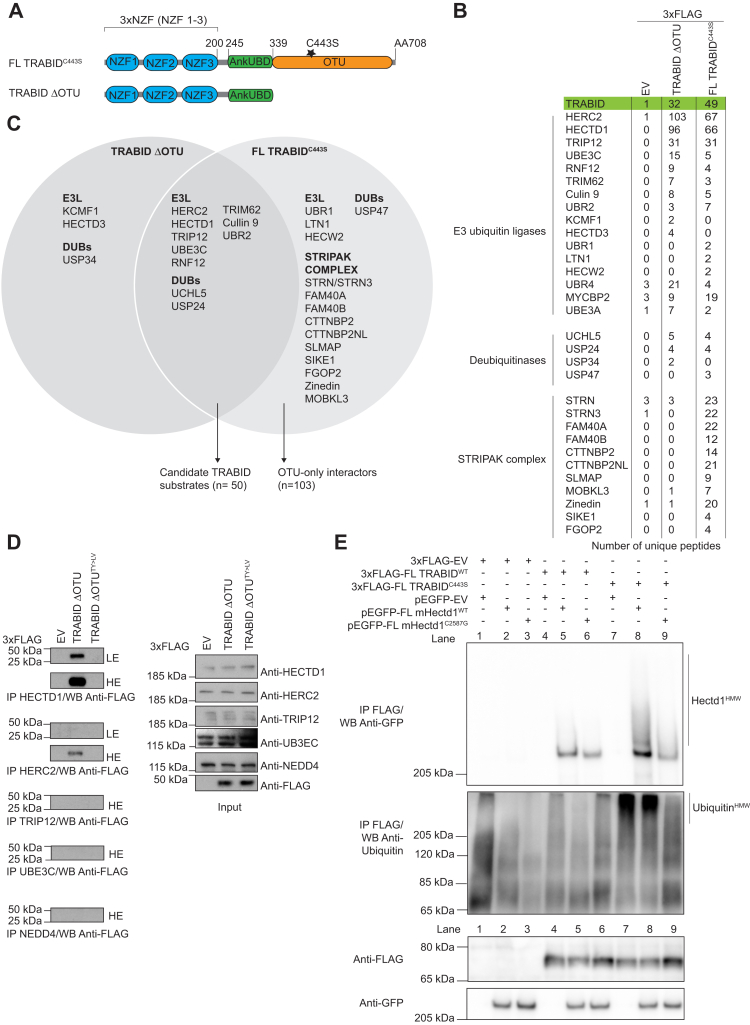
**TRABID catalytic dead interactomes reveal OTU-dependent and independent interactors.***A*, domain organization of the catalytic dead TRABID constructs used in this proteomics study, TRABID ΔOTU and Full-Length (FL) TRABID^C443S^. Key features include the three Npl14-zinc finger (NZF) ubiquitin binding domains (25, 32), the AnkUBD (28) and the OTU catalytic domain (24). *B*, pCMV-3xFLAG_EV, TRABID ΔOTU, or FL TRABID^C443S^ was transiently expressed in HEK293ET. Following immunoprecipitation (IP) with FLAG M2 magnetic beads, samples were subjected to SDS-PAGE and gel slices were cut and analyzed on an Orbitrap velos (IP experiments were n = 1 for each of the three plasmids). A selection of candidates TRABID interactors is shown, with the number of unique peptides. Note that STRIPAK components were previously reported, and our data now identifies the OTU as the domain on TRABID mediating interaction with components of this complex (39). From a list of 2225 proteins, only those with zero unique peptide in the empty vector condition were shortlisted for further analysis (n = 208). We also included in this list seven proteins that did not meet this criterion, on the basis that these were part of the STRIPAK complex (STRN, STRN3, Zinedin), or that they were E3 ligases (HERC2, MYCBP2, UBR4, UBE3A), bringing the working list to n = 215 (Table S1). We applied a cutoff of at least two unique peptides to generate the OTU-specific list (n = 103), the TRABID ΔOTU-specific list (n = 23), and a candidate substrate list common to TRABID ΔOTU or FL TRABID^C443S^ (n = 50). *C*, Venn diagram showing selected candidate interactors for each and also for both catalytic dead TRABID constructs. Given the role of the NZF domains in ubiquitin binding, the list common to both constructs likely represents substrates of TRABID DUB activity. *D*, validation of HECTD1 as TRABID interactor. pCMV-3xFLAG_EV, TRABID ΔOTU, or FL TRABID ΔOTU^TY>LV^ was transiently expressed in HEK293ET cells. Twenty-four hours following transfection, cell lysates were incubated with Dynabeads magnetic beads coupled with antibodies for the indicated HECT ligases. IP samples and input were analyzed by western blot using the indicated antibodies. NEDD4 was not found in our interactome and was used as negative control. TRIP12 could barely be detected, which is not surprising given its reported nuclear localization. Note that the membrane had to be overexposed in order to detect 3xFLAG TRABID ΔOTU in the HERC2 IP. *E*, immunoprecipitation assays showing the interaction between ectopically expressed TRABID and HECTD1. pCMV-3xFLAG_Ev, FL TRABID^WT^, or FL TRABID^C443S^ was transiently expressed together with either pEGFP-Ev, pEGFP-FL mouse Hectd1^WT^, or pEGFP-FL mouse Hectd1^C2587G^ as indicated. FLAG M2 beads were used to immunoprecipitate FLAG-tagged plasmids. Note the enrichment of higher-molecular-weight species of Hectd1 (Hectd1^HMW^) in the condition where catalytic dead TRABID has been coexpressed (IP FLAG/WB GFP, lane 8 *versus* 5). Hectd1^HMW^ is markedly reduced in the catalytic dead-Hectd1 condition (IP FLAG/WB Ubiquitin, lane 9 *versus* 8). TRABID catalytic dead traps high-molecular-weight ubiquitin species (IP FLAG/WB Ubiquitin; Lane 8 *versus* 5), unless catalytic dead Hectd1 is coexpressed (IP FLAG/WB Ubiquitin, lane 8 *versus* 9). This suggests that TRABID DUB activity regulates ubiquitin chains on Hectd1. Reciprocal IPs with FLAG-tagged FL mHectd1 and GFP-tagged FL TRABID yielded similar observation (Fig. S1*E*).

TRABID is highly tuned for recognizing and processing K29 and K33 ubiquitin linkages. Yet, most cellular mechanisms reported to date have implicated a K63-specific DUB activity. To explore this further, we analyzed the interactome of two catalytic dead TRABID constructs following transient expression in HEK293ET cells (Fig. 1, *A*–*C*). Both TRABID^C443S^ and TRABID ΔOTU are catalytically inactive but can still efficiently trap ubiquitin through the ubiquitin binding property of the NZF domains (32, 33). To validate this, we used immunoprecipitation assays and a cellular puncta formation assay, which we previously used as a readout to visualize polyubiquitin trapped by catalytic dead TRABID in cells (Fig. S1, *A*–*C*) (28, 31, 32). Point mutations (TY to LV) in each of the NZF domains reduced ubiquitin trapping as shown by a reduced ubiquitin smear and complete loss of puncta formation (Fig. S1, *B* and *C*, respectively) (25, 32). Together, this data confirms that loss of TRABID DUB activity results in the trapping of ubiquitinated species, visualized as puncta in cells, and this requires functional NZFs. Although a minimal construct consisting of TRABID NZF 1 to 3 (AA1-200) should in theory suffice to trap ubiquitin, such a construct was less efficient in both our trapping immunoprecipitation and puncta formation assays. This likely reflects the additional contribution of AnkUBD in enhancing ubiquitin trapping (28).

By comparing the interactome of TRABID^C443S^ to that of TRABID ΔOTU, we aimed to differentiate between *bona fide* substrates and interactors since it is the NZF domains, and not the OTU domain, that mediate substrate recognition. 3xFLAG_empty vector (Ev), 3xFLAG_full-length-TRABID^C443S^ and 3xFLAG_TRABID ΔOTU were transiently expressed in HEK293ET cells, immunoprecipitated with FLAG M2 magnetic beads, followed by LC-MS/MS analysis on an Orbitrap (Fig. S1*D*). The initial list included 2225 proteins with at least two unique peptides in either TRABID interactomes and zero unique peptide in the empty vector control condition (Table S1). Our working list included 23 proteins exclusive to 3xFLAG_TRABID ΔOTU and 103 proteins exclusive to full-length 3xFLAG_TRABID^C443S^. Nearly all the components of the STRIPAK complex including Striatin 3, CTTNBP2/CTTNBP2L, SLMAP, and FAM40A/FAM40B were identified in the 3xFLAG-full-length-TRABID^C443S^, but not the 3xFLAG_TRABID ΔOTU condition. Although this kinase/phosphatase complex had already been identified in the original full-length TRABID^WT^ interactome, our data now show that the interaction between STRIPAK and TRABID is primarily mediated by the catalytic OTU domain rather than the ubiquitin binding properties of TRABID NZF (Fig. 1, *B* and *C*) (39).

**Figure 2:**
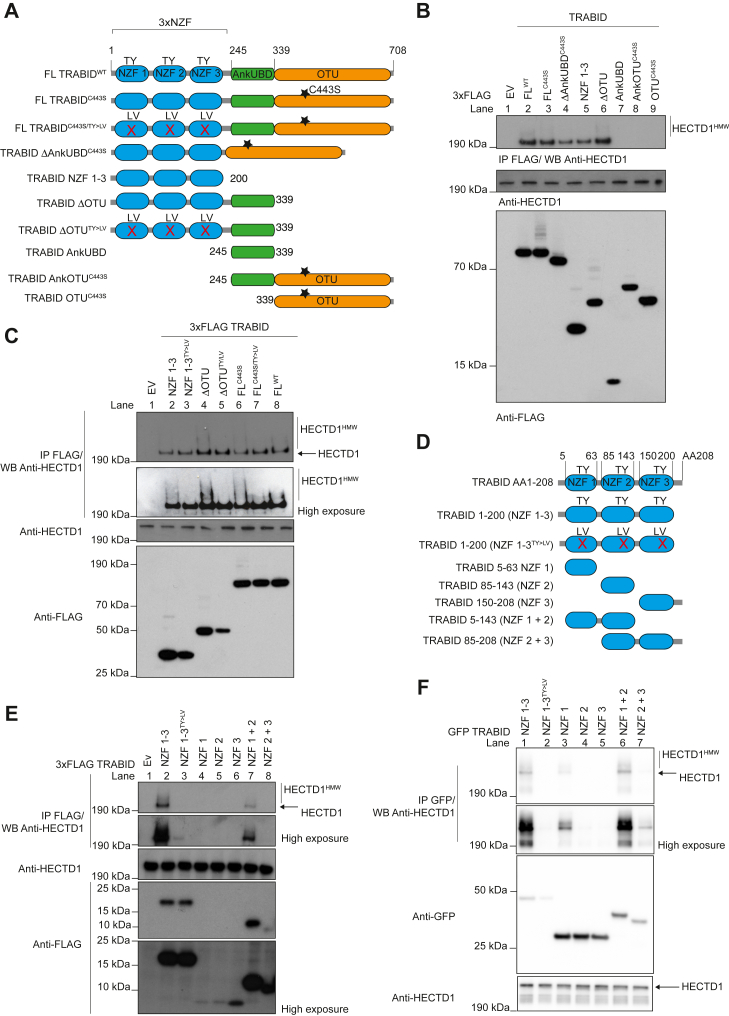
**Mapping of TRABID-HECTD1 interaction.***A*, domain organization of TRABID constructs used to map the interaction with HECTD1. The point mutation C443S abrogates TRABID DUB activity while TY>LV mutations in each of the three NZFs abrogate their ability to bind ubiquitin. *B*, immunoprecipitation assay using the indicated 3xFLAG-TRABID deletion constructs, showing that TRABID 1 to 200 (NZF 1–3) is key for its interaction with endogenous HECTD1. Constructs were transiently expressed for 24 h in HEK293ET cells prior to immunoprecipitation with FLAG M2 beads, followed by western blot analysis. *C*, immunoprecipitation assays performed as in B), using either catalytic dead-constructs (Lanes 2, 4, 6), their cognate triple NZF mutant (TY>LV) (Lanes 3, 5, 7) or FL TRABID^WT^ (Lane 8). Note that HECTD1 higher-molecular-weight species (HECTD1^HMW^) could be detected in all conditions apart from 3xFLAG-Ev and 3xFLAG-TRABID^WT^ IPs. High exposure (HE) of the membrane following ECL detection was necessary to show these HMW species. Note that residues mutated in TRABID NZFs (TY>LV) affect their ability to bind ubiquitin but not their fold, and all three NZFs were mutated in these constructs (25, 32). *D*, domain organization of constructs containing either individual or multiple NZFs as indicated. The NZF triple mutant is also depicted. 3xFLAG-TRABID NZF (*E*) or pEGFP-TRABID NZF (*F*) constructs were used to identify the minimal region that mediated interaction with endogenous HECTD1. Constructs were transiently transfected as in *B* and *C*. Depending on whether one or two NZF were included, the construct expressed differently, hence why we also tried GFP-tagged constructs, which improved the expression of individual NZFs (*F*). High exposure of blots is provided to better visualize the different level of expression of these constructs and also to show that NZF 1 is the minimal domain mediating HECTD1 interaction (*F*, lane 3). Note the clear reduction in signal in the TRABID NZF 1 to 3^TY>LV^ (*E*, lane 3 *versus* 2; *F*, lane 2 *versus* 1), which is in line with C (Lanes 3, 5, 7 *versus* 2, 4, 6, respectively).

We also identified 50 proteins common to both 3xFLAG_TRABID ΔOTU and 3xFLAG_ FL TRABID^C443S^ (Table S1) and therefore reasoned that this list likely represented direct substrates of TRABID DUB activity. DUBs often exist as part of protein complexes, which can include other DUBs as well as E3 ubiquitin ligases, and we found both classes of enzymes in our candidate substratome list (Fig. 1, *B* and *C*; Table S1) (40). The identification of the E3 ubiquitin ligase HECTD1 as a candidate substrate of TRABID had been previously suggested (33, 39). However, the nature of this interaction remained puzzling given that HECTD1 has been suggested to assemble K63-linked chains, while TRABID preferentially recognizes and cleaves K29 and K33 linkages. Therefore, we further explored the TRABID-HECTD1 interaction in order to reconcile and also expand on these observations. First, we determined whether the trapping of E3 ubiquitin ligases by catalytic dead TRABID was dependent on ubiquitin. For this, we immunoprecipitated the indicated endogenous HECT ligases from HEK293ET cells overexpressing 3xFLAG_TRABID ΔOTU or 3xFLAG_TRABID ΔOTU^TY>LV^, a ubiquitin binding deficient mutant (Figs. 1*D* and 2*A*). We chose HECTD1, TRIP12, UBE3C, HERC2, since these had the highest number of unique peptides identified in our pull-down LC/MS-MS experiments. NEDD4 was used as negative control since it was not identified in any of our interactome studies. Endogenous HECTD1 and HERC2 could both be immunoprecipitated by the trapping construct 3xFLAG_TRABID ΔOTU, and this interaction required functional NZFs since the TY>LV mutant abrogated these interactions (Fig. 1*D*). In contrast, we could not validate TRIP12 and UBE3C as interactors/substrates trapped by catalytic dead TRABID although this could reflect the abundance and/or expression pattern of these E3s. For instance, TRIP12 has been shown to primarily localize to the nucleus, and its low recovery in our immunoprecipitation assay could be due to the lysis buffer used.

### HECTD1 is a substrate of TRABID DUB activity

We next used immunoprecipitation assays to show that FL TRABID^C443S^ indeed traps higher-molecular-weight species corresponding to ectopically expressed full-length mouse Hectd1 (Fig. 1*E*, lane 8, Hectd1^HMW^). In contrast, in the condition where TRABID is active (Fig. 1*E*, lane 5), or if catalytic dead FL mouse Hectd1^C2587G^ is used (Fig. 1*E*, lane 9), Hectd1^HMW^ species were markedly reduced. This suggests that catalytic dead TRABID traps higher-molecular-weight species of Hectd1 and that these species are dependent upon the ubiquitin ligase activity of Hectd1. We obtained similar data when switching 3xFLAG and EGFP tags around (Fig. S1*E*). Conjointly, this indicates that TRABID regulates Hectd1 autoubiquitination in cells. Importantly, these observations held true for endogenous HECTD1 (Fig. 2). By using deletion constructs in immunoprecipitation experiments, we found that TRABID NZF 1 to 3 is required for its interaction with endogenous HECTD1 (Fig. 2, *A* and *B*). In fact, with increased exposure of the membrane, we were able to detect endogenous HECTD1^HMW^ species trapped by TRABID ΔOTU, FL-TRABID^C443S^, and to a lesser extent TRABID NZF 1 to 3 (Fig. 2*C*, lanes 2, 4, 6). Importantly, HECTD1^HMW^ could not be trapped by the corresponding NZF TY>LV mutants, nor by active/wild-type TRABID (Fig. 2*C*, lanes 3, 5, 7 and 8). Further mapping using 3xFLAG-tagged and EGFP-tagged TRABID NZF constructs narrowed down the minimal domains involved as NZF 1 + 2 (Fig. 2*D-F*). Since individual TRABID NZF constructs expressed better when GFP tagged, we repeated the immunoprecipitation assay shown in Figure 2*E* with these constructs and identified NZF 1 has the minimal domain mediating interaction with endogenous HECTD1 (Fig. 2*F*, lane 3). This is important since TRABID NZF 1 is a K29/K33-specific UBD (29, 30, 31).

**Figure 3:**
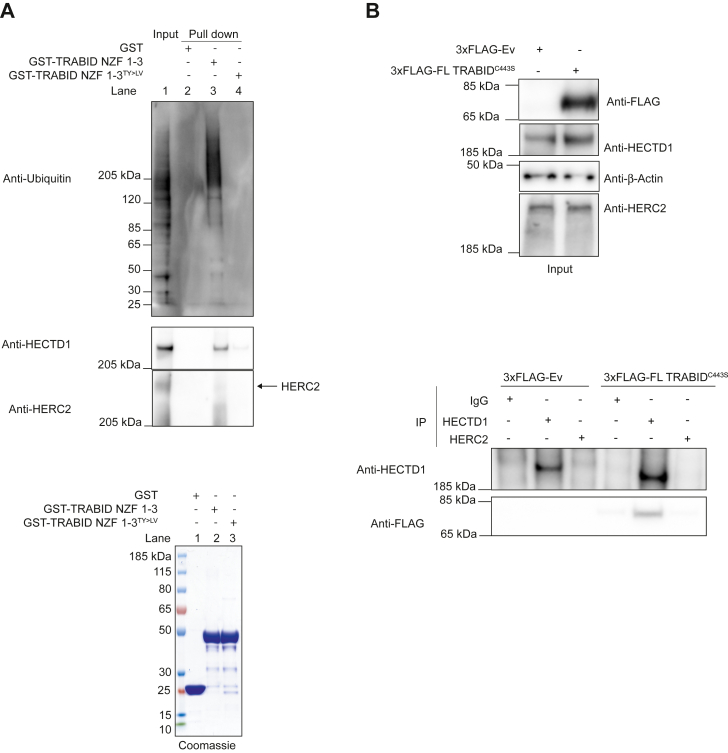
**TRABID NZFs are required for trapping ubiquitin and endogenous HECTD1.***A*, GST pull-down assays were carried using 20 μg of GST, GST-TRABID NZF 1 to 3, or GST-TRABID NZF 1 to 3^TY>LV^. Loss of ubiquitin binding through TY>LV mutations abrogates binding with endogenous ubiquitin and endogenous HECTD1. *B*, immunoprecipitation assays showing that endogenous HECTD1, but not HERC2, binds TRABID. This data also shows that HECTD1 and HERC2 do not interact, at least in this assay. Four micrograms of either HECTD1 or HERC2 antibody was coupled to Dynabeads magnetic beads and incubated with lysates of HEK293T cells expressing either 3xFLAG-Ev or 3xFLAG-FL TRABID^C443S^. Following four washes with lysate buffer, 2× LDS/100 mM DTT was added to the beads, and samples were heated for 5 min at 95 °C followed by western blot analysis. Input samples are shown on the *top panel* and IPs on the *lower panel*.

Although we attempted to validate the interaction between endogenous TRABID and endogenous HECTD1, none of the four TRABID antibodies that we tried could detect endogenous TRABID (Data not shown). As an alternative approach, we further validated this interaction using recombinantly expressed GST-tagged TRABID NZF 1 to 3 as bait (Fig. 3*A*). GST-TRABID NZF 1 to 3, but not its ubiquitin binding deficient mutant GST-TRABID NZF 1 to 3^TY>LV^ nor GST alone, could enrich for high-molecular-weight ubiquitin chains as well as endogenous HECTD1 (Fig. 3*A*, lane 3). Since HERC2 was also found to potentially interact with catalytic dead TRABID, we further validated the IP data shown in Figure 1*D* more stringently, by including an IgG control IP. Rabbit IgG, HECTD1 or HERC2 antibodies were coupled to magnetic Dynabeads and used in immunoprecipitation assays using lysates from HEK293ET overexpressing either 3xFLAG_FL TRABID^C443S^ or an empty vector control (Fig. 3*B*). Although immunoprecipitation of endogenous HECTD1 led to a clear detection of 3xFLAG_FL TRABID^C443S^, we observed a similar signal between the IgG control IP and the endogenous HERC2 IP. This is perhaps not surprising given that the membrane had to be overexposed in Figure 1*D* to enable HERC2 detection. GST-TRABID NZF 1 to 3 pull-down showed a faint signal for HERC2 further suggestive of a very weak interaction (Fig. 3*A*).

### HECTD1 assembles K29 and K48-linked ubiquitin chains

DUBs and E3s have been shown to function as enzyme pairs, either regulating a particular cellular process, each other’s stability, and/or activity (40). For example: the DUB BRCC36 together with the RING E3 ligase BRCA1 regulates the DNA damage response; the RBR E3 ligase Parkin and the DUB USP30 contribute to mitophagy; the DUB Ubp2 can impede yeast growth by antagonizing the function of the HECT E3 Rsp5; the DUB FAM/USP9x interacts with and stabilizes SMURF1 and this impacts on cell motility (41, 42, 43). Therefore, we further explored the molecular basis for the TRABID-HECTD1 interaction and hypothesized that HECTD1 could assemble ubiquitin chains that are subject to TRABID DUB activity. This implied that HECTD1 was modified, perhaps through autoubiquitination, with K29- and/or K33-linked chains given these are preferentially recognized by TRABID NZF 1 (29, 30, 31).

**Figure 4:**
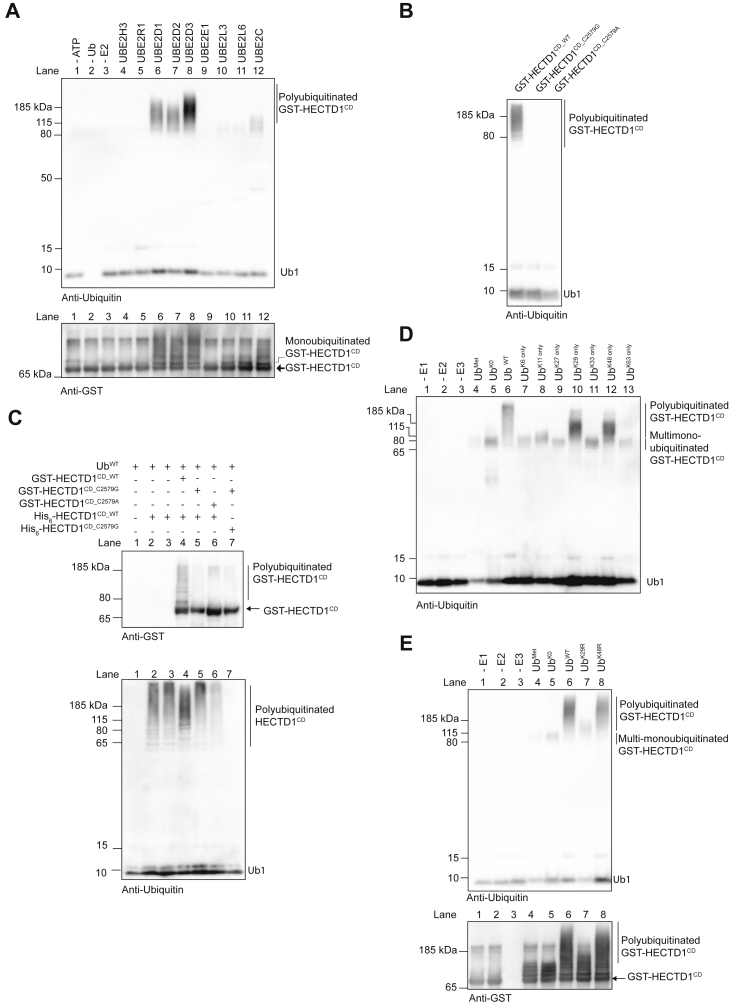
**HECTD1 catalytic HECT domain autoubiquitinates.***A*, *in vitro* autoubiquitination assay performed as previously described (46). GST-HECTD1^CD^ (Catalytic domain of human HECTD1, boundaries AA2129-End) was incubated in the presence of E1 (UBE1), ubiquitin, and the indicated E2 conjugating enzymes. Reactions without ATP, ubiquitin, or E2 were included as controls. Reactions were stopped after 3 h at 30 °C by the addition of 2X SDS/100 mM DTT and analyzed by western blotting using (*Top panel*) and anti-GST (*Lower panel*) antibodies. The ubiquitin smear observed reflects the autoubiquitination of GST-HECTD1^CD^ and was used as readout for ubiquitin ligase activity. *B*, autoubiquitination assays were carried out as in *A*, using UBE2D1 and GST-HECTD1^WT^ or the indicated catalytic dead constructs, GST-HECTD1^C2579G^ or GST-HECTD1^C2579A^. *C*, *in vitro* assay showing His_6_-HECTD1^CD^ cannot ubiquitinate catalytic dead GST-HECTD1^CD^ (C2579A or C2579G). Therefore, the observed autoubiquitination of GST-HECTD1^WT^ occurs in *cis*. *D*, *in vitro* autoubiquitination carried out as in *A* using GST-HECTD1^CD^ and UBE2D1, for 3 h at 30 °C, but using Konly ubiquitin mutants (*i.e.*, only the indicated lysine residue has not been mutated to arginine). In order to differentiate between multi-monoubiquitination events and polyubiquitination, Ub^Met^ and Ub^K0^ were used as controls. Additional control samples included a no E1, no E2, or no E3 reactions. Reactions were stopped as in *A* and analyzed by western blotting using an anti-ubiquitin antibody. *E*, *in vitro* autoubiquitination carried out as in *A* at 30 °C for 3 h, using GST-HECTD1^CD^, UBE2D1, wild-type ubiquitin, or the indicated Ub^K/R^ ubiquitin mutants (*i.e.*, only the indicated lysine is mutated to arginine). Reactions were stopped and analyzed as mentioned previously.

Determining the type and composition of ubiquitin chains on protein targets in cells has proved to be challenging. Therefore, we exploited the property of C-terminal HECT domains to autoubiquitinate *in vitro* in the absence of a substrate as a proxy for determining HECTD1 ubiquitin ligase specificity (Fig. 4) (44, 45). We previously identified UBE2D1, 2, and 3 as HECTD1’s cognate E2s, while UBE2L3 showed little if any cooperativity for polyubiquitin chain assembly (46). We now further expand this analysis to a set of E2s including UBE2H3, UBE2R1, UBE2E1, UBE2L6, and UBE2C (Fig. 4*A*). Although UBE2L3, UBE2L6, and UBE2C seemed competent in priming GST-HECTD1^CD^ with one ubiquitin molecule as shown by the monoubiquitinated GST-HECTD1^CD^ signal, there was weak HECTD1 ligase activity as indicated by a faint ubiquitin smear (Fig. 4*A*, lanes 10–12). This suggests that in contrast to UBE2Ds, which can initiate and elongate ubiquitin chains on HECTD1 (Fig. 4*A*, lanes 6–8), UBE2L3, UBE2L6, and UBE2C can only prime HECTD1 with a single ubiquitin moiety. In contrast, UBE2H3 and UBE2R1 showed no cooperation with HECTD1 ligase activity as (Fig. 4*A*, lanes 4 and 5). Specific E2-E3 cooperativity has been reported for other HECT ligases (47). The best example for E2-E3 cooperativity in cells is in the context of mitosis where UBE2C assembles short chains on mitotic cyclins prior to their elongation with K11-linked chains *via* UBE2S (48, 49, 50). A recent report further addressed the molecular basis for E2-E3 cooperativity in the context of ERAD RING E3 ligases Doa10 and Hrd1, which have distinct preferences for Ubc6 and 7 for priming, but not for chain elongation (51).

Some E2 conjugating enzymes have also been shown to drive ubiquitin transfer onto free lysine residues independently of E3 activity, and this is particularly relevant for substrates that contain a ubiquitin binding domain (52, 53). For instance, UBE2D3∼Ub reacts with free lysine and cysteine residues, whereas UBE2L3∼Ub only reacts with cysteines (54). Although this mechanism of E3-independent ubiquitin transfer is more relevant for RING E3 ligases, we nevertheless validated that the activity we observed in our assay was due to the catalytic HECT domain specifically. Indeed, a point mutation in the catalytic cysteine residue (C2579G or C2579A) abrogated HECTD1 ligase activity (Fig. 4*B*). To further ascertain that the observed HECTD1 ligase activity represents autoubiquitination, we next tested whether it occurred in *cis* or *trans*. For this, we tested whether wild-type His_6_-HECTD1^CD^ could ubiquitinate catalytic dead GST-HECTD1^CD (C2579G or C2579A)^. However, we found that GST-HECTD1^CD^ catalytic mutants were not polyubiquitinated in the presence of active His_6_-HECTD1^CD^ (Fig. 4*C*, Anti-GST blot, lanes 5 and 6). This indicates that autoubiquitination of HECTD1^CD^ occurs in *cis*.

We next used *in vitro* autoubiquitination assays and ubiquitin mutants to determine the type of ubiquitin chains preferentially synthesized by HECTD1 (Fig. 4*D*). Ubiquitin, either methylated at all Lys (*i.e.*, Ub^Met^) or mutated at all lysine residues into arginine (UbK^0^), was used as control to reveal multi-monoubiquitination events on GST-HECTD1^CD^. In contrast to ubiquitin WT, which showed the full extent of ubiquitin chains synthesized by HECTD1 (Fig. 4*D*, lane 6), both Ub^Met^ and Ub^K0^ showed distinct bands indicative of multi-monoubiquitination (Fig. 4*D*, lanes 4 and 5, respectively). Ubiquitination assays with ubiquitin K^only^ mutants, which have all, but the indicated lysine mutated to arginine, were then used to reveal HECTD1 specificity in terms of the linkages utilized to assemble ubiquitin chains. Using ubiquitin K27^only^, K33^only^, or K63^only^ mutants led to autoubiquitination patterns similar to the multi-monoubiquitination controls. In contrast, Ub^K29only^ and Ub^K48only^ showed ubiquitin smears corresponding to polyubiquitinated HECTD1 (Fig. 4*D*, lanes 10 and 12). However, neither Ub^K29only^ nor Ub^K48only^ recapitulated the full smear obtained with Ub^WT^, suggesting that HECTD1 might assemble mixed K29- and K48-linked ubiquitin chains (Fig. 4*D*, lanes 10 and 12 *versus* 6).

To further validate the requirement for K29 and K48 linkages for ubiquitin chain assembly by HECTD1, autoubiquitination assays were carried out using K/R ubiquitin mutants, which have only the indicated lysine residue mutated to arginine. In the presence of Ub^K29R^, ubiquitin chain formation was markedly reduced (Fig. 4*E*, lane 7 *versus* 6). In contrast, Ub^K48R^ had a marginal effect on the ubiquitin smear (Fig. 4*E*, lane 8 *versus* 6). This indicates that K29 rather than K48 is the likely prime linkage used by HECTD1 to assemble the core of the polyubiquitin chains we observed.

### Quantitative linkage analysis of ubiquitin chains assembled by HECTD1

**Figure 5:**
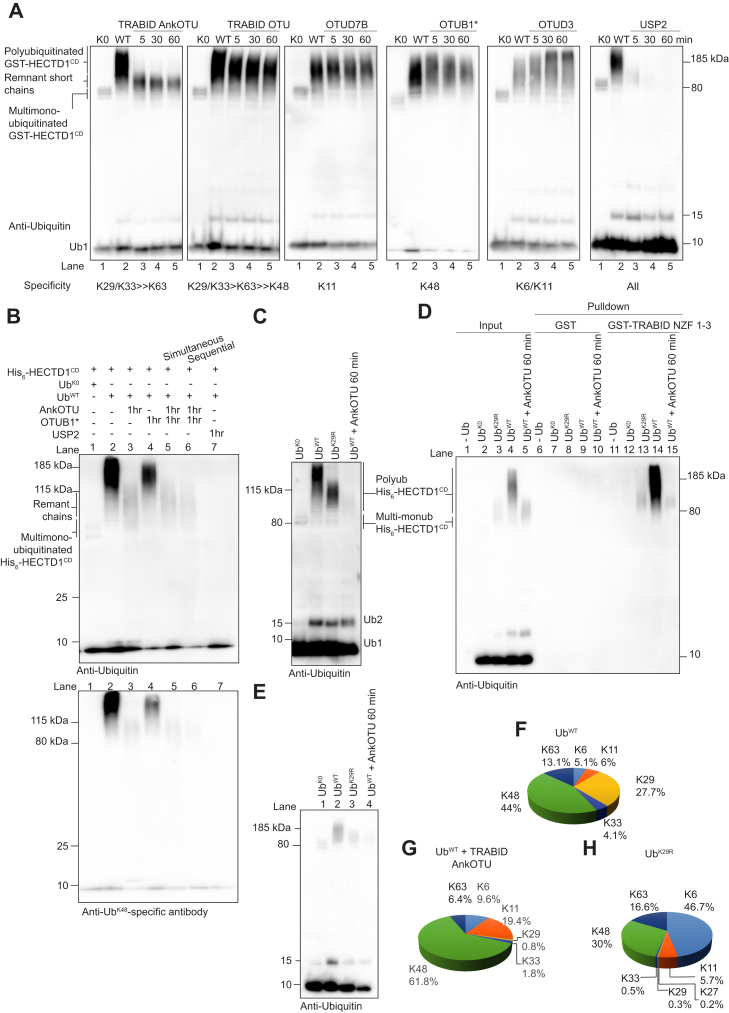
**HECTD1 assembles polyubiquitin chains containing K29 and K48 linkages.***A*, time-course UbiCREST assays were performed on polyubiquitinated GST-HECTD1^CD^ (55, 56). *In vitro* autoubiquitination was carried out using GST-HECTD1^CD^, ATP, UBE1, UBE2D1, ubiquitin K0 (in order to identify multi-monoubiquitination events, lane 1), or ubiquitin WT (Lanes 2–5). Following incubation for 3 h at 30 °C, reactions were terminated through the addition of 2 mU apyrase for 20 min prior to addition of TRABID AnkOTU (2.5 μM), TRABID OTU (2.5 μM), OTUD7B/Cezanne (1 μM), OTUB1^∗^ (2 μM), OTUD3 (2 μM) or USP2 (1 μM) for 5 min, 30 min, or 60 min. UbiCREST reactions were terminated at the indicated time points by addition of 2X LDS/100 mM DTT, resolved on a 4 to 12% SDS PAGE and analyzed by western blotting using an anti-ubiquitin antibody. Note that remnant ubiquitin smears (AnkOTU, lanes 3–5) are shorter than smears obtained with Ub^WT^ (Lane 2) but longer than those in the multi-monoubiquitination control reaction (Lane 1). *B*, double UbiCREST assay. *In vitro* autoubiquitination assays were carried out as in *A* but with His_6_-HECTD1^CD^ and incubated with 1 μM of AnkOTU (Lane 3), 2 μM of OTUB1∗ (Lane 4), or 2 μM of USP2 for 1 h at 30 °C (Lane 7), as indicated. AnkOTU and OTUB1∗ were also incubated at the same time and for 1 h (Lane 5) or sequentially (*i.e.*, AnkOTU was added for 5 min followed by OTUB1∗ for 1 h, lane 6). Reactions were stopped by addition of in 2X LDS/100 mM and analyzed by western blot using a ubiquitin (P4D1) antibody or an anti-UbK48-specific antibody. Note that the double UbiCREST does not lead to a reduction in the remnant ubiquitin signal that is obtained following AnkOTU treatment alone (Lane 3). Similar data was obtained with GST-HECTD1^CD^ (Fig. S2D). *C*, *in vitro* autoubiquitination assays were carried out in the presence of ATP, UBE1, UBE2D1, and His_6_-HECTD1^CD^ for 3 h at 30 °C. Ubiquitin wild type, Ub^K0^ (*i.e.*, control to show multi-monoubiquitination), or Ub^K29R^ was used, as indicated. Autoubiquitinated His_6_-HECTD1^CD^ obtained with Ub^WT^ was incubated with of 2.5 μM of TRABID AnkOTU for 1 h at 30 °C, stopped by the addition of 2xLDS/100 mM DTT and analyzed by western blotting using an anti-ubiquitin antibody. *D*, GST pull-down of His_6_-HECTD1^CD^ either unmodified (Lanes 1, 6, 11), or modified with multi-monoubiquitination (Lanes 2, 7, 12), short chains (Lanes 3, 8, 12), or long chains (Lanes 4, 9, 14). Reactions showing remnant chains, which remain following TRABID AnkOTU treatment (Lanes 5, 10, 15), are also shown. GST pull-down was carried out as previously described (26). Briefly, GST or GST-TRABID^NZF 1–3^ was coupled to glutathione magnetic beads for 1 h at RT. Beads were washed in pull-down buffer + BSA and then added to each of the *in vitro* ubiquitin reactions. Following overnight incubation on at 4 °C beads were washed with pull-down buffer five times. Beads were finally resuspended in 2X LDS/100 mM samples buffer and analyzed by western blotting using an anti-ubiquitin antibody and also by silver stain to monitor protein loading (Fig. S3). Autoubiquitination assays shown in *E* were analyzed by Ubiquitin-AQUA proteomics for Ub^WT^ (*F*), Ub^WT^ followed by TRABID AnkOTU incubation (*G*), or Ub^K29R^ (*H*) (Fig. S4; Table S2).

To determine the linkage composition of ubiquitin chains assembled by HECTD1 with more precision, we carried out UbiCREST and ubiquitin-absolute QUAntification (Ubiquitin-AQUA) on *in vitro* autoubiquitinated GST-HECTD1^CD^ (Fig. 5) (15, 55, 56, 57). Polyubiquitinated GST-HECTD1^CD^ was incubated with the indicated linkage-specific DUBs for 5, 30, or 60 min, prior to western blot analysis with an anti-ubiquitin antibody. Polyubiquitinated GST-HECTD1^CD^ was efficiently deubiquitinated by the AnkOTU domain of TRABID, which suggests that HECTD1 assembles chains through K29 and/or K33 linkages, since these are the preferred TRABID substrates (Fig. 5*A*) (28). Interestingly, remnant short ubiquitin chains of size similar to those obtained with Ub^K29R^ (Fig. 4*E*, lane 7) were also detected following incubation with TRABID AnkOTU (Fig. 5*A*, lanes 3–5 *versus* 1). In line with previous data, TRABID OTU domain alone (*i.e.*, lacking the AnkUBD) showed limited activity (28). In order to further validate the linkage composition of chains assembled by HECTD1, we used DUBs of known specificity which have been particularly useful in order to determine the linkage composition of ubiquitin chains. These included the OTU DUBs OTUD7B/Cezanne (K11-specific), OTUD3_CD (cleaves K6/K11 linkages), the JAMM family member AMSH (K63-specific) and its more active AMSH∗ (improved activity toward K63 chains), and the proteasomal DUBs UCH37 (cleaves Met-1 and K48-specific linkages) and USP14 (cleaves K6/11/48/K63) (31, 56, 58, 59, 60, 61, 62). None of these DUBs were able to reduce the ubiquitin smear induced by HECTD1 (Fig. 5*A* and Fig. S2, *A* and *B*). In contrast, OTUB1∗, which has been engineered to have increased DUB activity compared with OTUB1, showed some activity toward autoubiquitinated HECTD1 (Fig. 5*A*, OTUB1∗ lanes 3–5 *versus* 2; Fig. S2*C* for His_6_-HECTD1^CD^). To explore the possibility that these chains might be of mixed composition, through K29 and K48 linkages, we incubated autoubiquitinated HECTD1^CD^ with both AnkOTU and OTUB1 (Fig. 5*B*). However, we observed similar data than for the AnkOTU alone treatment (Fig. 5*B*, lanes 5 and 6 *versus* 3). Although the remnant chains that remained following AnkOTU treatment can be fully processed by USP2, OTUB1∗ had no effect on these chains, which could suggest that they might be branched and therefore resistant to DUB cleavage (Fig. 5*B* for His_6_-HECTD1^CD^; Fig. S2*D* for GST-HECTD1^CD^) (55).

Having established that TRABID NZF 1 to 3 is required for binding HECTD1 in cells and that catalytic dead TRABID traps polyubiquitinated HECTD1, we then validated this data in the context of HECTD1’s newly identified ubiquitin ligase activity using a pull-down approach (Fig. 5, *C* and *D*; Fig. S3). For this, we used His_6_-HECTD1^CD^ instead of GST-HECTD1^CD^ since this construct yielded longer polyubiquitinated signals which were easier to visualize. Importantly, His_6_-tagged HECTD1^CD^ displayed similar ligase activity as the GST-tagged version, as shown by the marked reduction in ubiquitin chain smear in the presence of Ub^K29R^ or following UbiCREST assay with AnkOTU (Fig. 5*C*). Polyubiquitinated His_6_-HECTD1^CD^, generated *in vitro*, could be efficiently enriched for by GST-TRABID NZF 1 to 3 pull-down but not GST alone (Fig. 5*D*, lane 14 *versus* 9). As expected, there was no clear enrichment when using Ub^K0^ in the autoubiquitination reaction, further indicating that TRABID NZF 1 to 3 does not bind monoubiquitin (Fig. 5*D*, lane 12) (29, 31, 32). The short ubiquitin species generated by His_6_-HECTD1^CD^ using Ub^K29R^, as well as the short remnant chains obtained following TRABID AnkOTU treatment, were not efficiently enriched by TRABID NZF 1 to 3, suggesting that these chains might contain linkages with low binding affinity for NZF 1 to 3 (Fig. 5*D*, lanes 13 and 15).

To corroborate these findings, we next analyzed the chains assembled by GST-HECTD1^CD^ using Ubiquitin-AQUA (Fig. 5, *E*–*H*, Fig. S4, and Table S2). This revealed that in the presence of ubiquitin WT, K48-linked and K29-linked ubiquitin chains are the preferred linkages used by HECTD1 to assemble polyubiquitin, with 44% and 27.7%, respectively (Fig. 5*F*). In agreement with our UbiCREST data, K29 linkages almost entirely disappeared following incubation with TRABID AnkOTU (Fig. 5*G*). We also analyzed the remnant ubiquitin chains obtained with Ub^K29R^, which revealed that in the absence of K29, HECTD1 favors K6 to assemble short ubiquitin chains (46.7% with Ub^K29R^ *versus* 5.1% with Ub^WT^) (Fig. 5*H*). Interestingly, the HECT ligase UBE3C assembles ubiquitin chains through K29 (47%) and K48 (48%) linkages. However, in the context of phosphorylation of ubiquitin at Ser 20, UBE3C utilizes K48 (79%) and K6 (6%) (63). This, together, with our data using Ub^K29R^, indicates that certain lysine residues on ubiquitin are prioritized by HECT ligases to assemble chains (64). Linkage prioritization can be regulated by phosphorylation, in the case of UBE3C, and it will be interesting to determine which posttranslational modifications on ubiquitin might affect HECTD1 specificity and how.

### HECTD1 and UBE3C assemble distinct K29-containing ubiquitin chains

**Figure 6:**
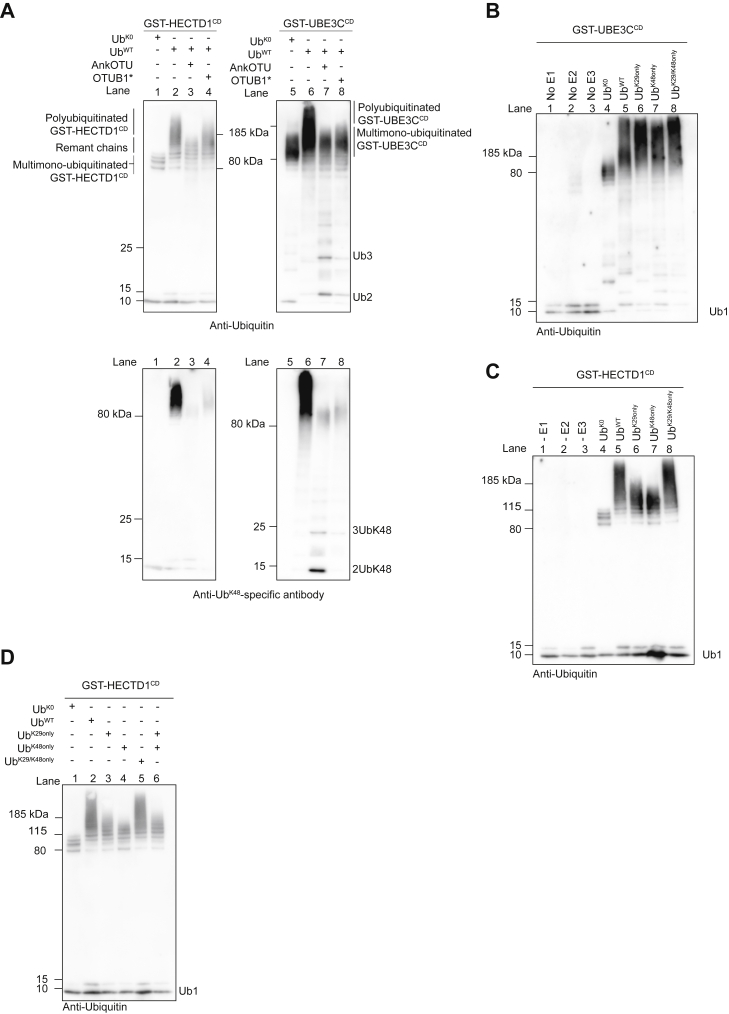
**UBE3C and HECTD1 assemble K29/K48-linked ubiquitin chains of different topology.***A*, *in vitro* autoubiquitination assays were carried out with GST-HECTD1^CD^ and GST-UBE3C^CD^ as in Figure 4. Following incubation for 3 h at 30 °C, reactions were terminated through the addition of 2 mU apyrase for 20 min prior to addition of TRABID AnkOTU (1 μM) or the K48-specific DUB OTUB1∗ (2 μM). Reactions were analyzed by western blotting using an anti-ubiquitin (*Top panel*) or a Ub^K48^-specific antibody (*Lower panel*). Note that as in Figure 5, remnant ubiquitin smears were observed for HECTD1 (Lane 3 *versus* lane 2 and 1) following AnkOTU treatment, but not for UBE3C (Lane 7 *versus* 6 and 5). Free ubiquitin chains (Ub2 and Ub3) were also detected following AnkOTU treatment of UBE3C polyubiquitinated species. Incubation of autoubiquitination reactions with OTUB1∗ had a more dramatic effect for UBE3C, which resulted in a ubiquitin smear similar to that obtained with Ub^K0^ and which indicates multi-monoubiquitination (Lane 8 *versus* 6). In contrast, OTUB1∗ had a more modest effect on the autoubiquitination smear produced with either GST-HECTD1^CD^ (Lane 4 *versus* 2) or His_6_-HECTD1^CD^ (Fig. S2*C*). Similar data was obtained when analyzing reactions with LI-COR IRDye antibodies (Fig. S5*C*). Probing reactions with an anti-Ub^K48^-specific antibody revealed that AnkOTU treatment released free Ub^K48^-linked ubiquitin chains, which were readily detected for UBE3C (*Lower panel*, lane 7 *versus* 3). In contrast, treatment with OTUB1∗ yielded virtually no such free chains, in line with its K48 DUB activity (Lane 8 *versus* 7). To establish whether the ubiquitin chains assembled through K29 and K48 had the same topology, we expressed and purified a Ub^K29/K48only^ mutant in E.coli and carried out *in vitro* autoubiquitination using either GST-UBE3C^CD^ (*B*) or GST-HECTD1^CD^ (*C*). Note the ability for UBE3C to assemble long chains irrespective of whether Ub^WT^ or the Ub^K^^29only^ or Ub^K48only^ is used (Lanes 6 and 7 *versus* 5). In contrast, HECTD1 could only assemble long chains in the presence of ubiquitin with both K29 and K48 available (Lanes 8 and 5 *versus* 7 and 6). *D*, to further establish that these represent branched rather than mixed chains, we repeated the reactions shown in C alongside a reaction containing both Ub^K29only^ and Ub^K48only^ proteins. This reaction has the potential to form heterotypic, but not branched, linkages. However, the chains assembled were comparable with those obtained with either of the Ub^K29only^ and Ub^K48only^ mutant alone, indicating the requirement for branching (Lane 6 *versus* 4 and 3).

UBE3C was the first and until now the only HECT ligase shown to assemble ubiquitin chains through both K29 and K48 linkages (45). We next set out to compare whether these E3s might both use these linkages to assemble chains with the same architecture. *In vitro* autoubiquitination reactions were performed with either GST-HECTD1^CD^ or GST-UBE3C^CD^ (catalytic HECT domain of HECTD1 and UBE3C, respectively) followed by treatment with AnkOTU or OTUB1∗ (Fig. 6*A*, Fig. S5, *A*–*C*). As previously shown, TRABID AnkOTU treatment resulted in short remnant ubiquitin chains for HECTD1 (Fig. 6*A*, lane 3 *versus* 1; Fig. S5*C*, lane 3 *versus* 1). In contrast, the same treatment resolved the polyubiquitinated smear produced by UBE3C down to the monoubiquitinated species signal (Fig. 6*A*, lane 7 *versus* 5; Fig. S5*C*, lane 6 *versus* 4). Side-by-side comparison of HECTD1^CD^ and UBE3C^CD^ also revealed that OTUB1∗ has a more pronounced effect on UBE3C, reducing its ubiquitin smear down to the monoubiquitination signal, compared with HECTD1^CD^ where a longer smear remained following OTUB1∗ treatment (Fig. 6*A*, lane 4 *versus* 1 for HECTD1^CD^; lane 8 *versus* 5 for UBE3C^CD^). The effect of OTUB1∗ on polyubiquitinated species was more readily observed when probing these reactions with a K48-specific antibody. In summary, this data suggests that HECTD1 and UBE3C both use K29 and K48 to assemble ubiquitin chains of different topologies. This is further emphasized by the detection of free ubiquitin chains, in particular dimers and trimers, which were readily detected following TRABID AnkOTU treatment of UBE3C but not HECTD1 (Fig. 6*A*, lower panel; Fig. S5*C*).

### HECTD1 ligase activity assembles ubiquitin chains through branched K29/K48 linkages

In order to determine whether branching was required as part of HECTD1 ligase activity to assemble K29/K48 chains, we expressed and purified an untagged Ub^K29/K48only^ mutant and used it in autoubiquitination assays (Fig. 6, *B*–*D*; Fig. S5*D*). We first compared GST-HECTD1^CD^ and GST-UBE3C^CD^ in their ability to assemble the full extent of ubiquitin chains that is seen with Ub^WT^, but in the presence of Ub^K29only^, Ub^K48only^, or Ub^K29/K48only^. UBE3C was able to use any of these Ub-only mutants to produce a full smear (Fig. 6*B*, lanes 6–8 *versus* 5). In contrast, when using either Ub^K29only^ or Ub^K48only^, HECTD1 could only assemble ubiquitin chains that were shorter than those obtained with Ub^WT^, in line with data shown in Figure 4*D* (Fig. 6*C*, lanes 6 and 7 *versus* 5). Strikingly, when using Ub^K29/K48only^, GST-HECTD1^CD^ was able to recapitulate the full activity seen with Ub^WT^ (Fig. 6*C*, lane 8 *versus* 5). To show that this activity for HECTD1 reflects branching rather than heterotypic mixed chains, we repeated the assay and included a reaction that contained both Ub^K29only^ and Ub^K48only^ (Fig. 6*D*). This produced a ubiquitin smear that was reminiscent of what we obtained with either Ub^K29only^ or Ub^K48only^ (Fig. 6*D*, lane 6 *versus* 3 and 4). Together, this data strongly indicates that the full ligase activity of HECTD1 relies on the ability to utilize K29 and K48 linkages on the same ubiquitin molecule, at least *in vitro*. In contrast, while UBE3C can use these same linkages to assemble chains, it does not appear to be reliant on branching. This data also could imply that the remnant chains observed following treatment with AnkOTU or OTUB1∗ represent branching linkages that cannot be efficiently processed by these DUBs.

Previous work established UBE3C’s preference for assembling K29 ubiquitin chains within mixed/branched chains (29, 65, 66, 67). The viral OTU (vOTU) of the Crimean Congo hemorrhagic fever virus (CCHFV), which cleaves all but Met-1 and K29-linked diubiquitin, has been useful to generate pure K29-linked chains for crystallography studies (68). We therefore used vOTU to test whether it could remove all but K29 linkages and provide further insights on the chains assembled by HECTD1 and UBE3C (29, 31, 55, 68, 69). In our assay, high concentration of vOTU fully resolved all the ubiquitin smears of both E3s, indicating that vOTU has the potential to cleave the remnant chains we previously observed in a similar fashion to USP2 (Fig. S6*A*; Fig. 5*A*). We next used Ub^K29only^ chains to titrate the concentration of vOTU so that it would not cleave K29-linked chains (Fig. S6*B*, lanes 4 and 11) (63). At this lower concentration, vOTU showed some weak activity toward the chains produced by HECTD1 and UBE3C when using Ub^K29/K48only^ (Fig. S6*C*). However, high-molecular-weight species could still be detected with the K48-specific antibody indicating incomplete digestion of this linkage by vOTU (Fig. S6*C*, upper panel, lane 4 *versus* 3; lane 10 *versus* 9). In summary of this data, treatment with vOTU did not provide further insights on the assembly of K29/K48 chains. In line with previous data obtained using Ub^WT^, free K48-linked ubiquitin chains were also released following AnkOTU treatment of polyubiquitinated UBE3C^CD^, when using Ub^K29/K48only^ (Fig. 6*A* for Ub^WT^; Fig. S6*C*, lane 11 for Ub^K29/K48only^).

**Figure 7:**
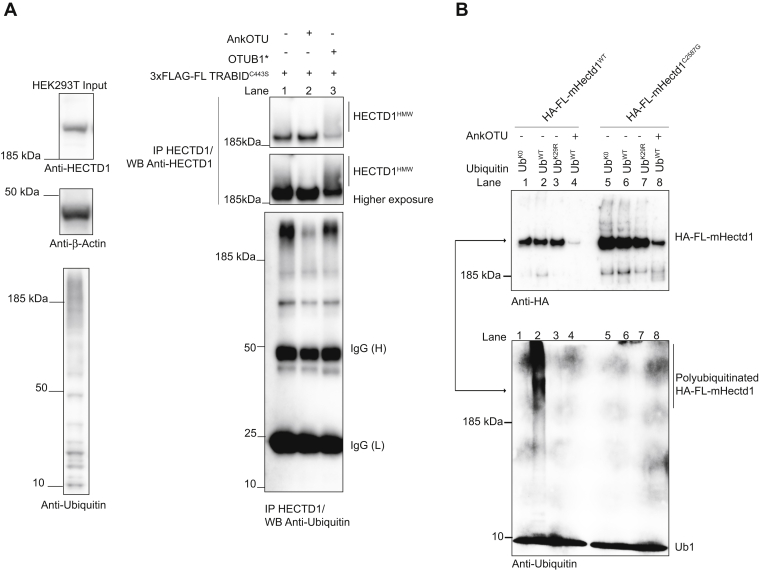
**Ubiquitin chains modifying full-length endogenous HECTD1 are processed by TRABID DUB activity.***A*, catalytic dead TRABID (3xFLAG TRABID^C443S^) was transiently overexpressed in HEK293T cells in order to trap polyubiquitinated endogenous HECTD1 (*i.e.*, HECTD1^HMW^ species). Following immunoprecipitation with FLAG M2 beads, beads were either untreated (*right panel*, lane 1) or treated with 1 μM of TRABID AnkOTU (*right panel*, lane 2), or 2 μM of OTUB1∗ (*right panel*, lane 3). Reactions were stopped by addition of 2X LDS/100 mM DTT and analyzed by western blot with the indicated antibodies. A higher exposure of the membrane is shown to facilitate the visualization of HECTD1^HMW^ species. TRABID DUB activity reduces HECTD1^HMW^ species as well as the overall level of trapped polyubiquitin chains (Lane 2 *versus* 1). In contrast, OTUB1∗ treatment has minimal effect on HECTD1^HMW^ (IP HECTD1/WB Anti-HECTD1, lane 3 *versus* 1) or on trapped polyubiquitin chains (IP HECTD1/WB Anti-Ubiquitin, lane 3 *versus* 1). *B*, *in vitro* autoubiquitination assay using HA-FL-mouse-Hectd1^WT^ or catalytically dead (HA-FL-mouse-Hectd1^C^^2587G^). HA-FL-mHectd1^WT^ or HA-FL-mHectd1^C2587G^ was transiently expressed in HEK293ET cells, immunoprecipitated using Pierce^TM^ anti-HA magnetic beads, eluted with an HA peptide, and used in autoubiquitination assays. Similar to the data obtained with GST-HECTD1^CD^, Ub^K29R^ does not support the full ligase activity of full-length mouse Hectd1 (Lane 3 *versus* 2). Furthermore, chains produced with Ub^WT^ are readily processed by TRABID AnkOTU (Lane 4 *versus* 2). HA-FL-mHectd1^C2587G^ was used as control and showed no autoubiquitination activity in this assay (Lane 6 *versus* 2).

Having shown that the catalytic domain of HECTD1 preferentially assembles ubiquitin chains through branching at K29 and K48, we next attempted to replicate this in cells in the context of full-length endogenous HECTD1. For this, we used 3xFLAG_FL TRABID^C443S^ to trap endogenous HECTD1^HMW^ species. Following immunoprecipitation using an anti-HECTD1 antibody coupled to Dynabeads, samples were left untreated or were incubated with either AnkOTU or OTUB1∗ for 1 h at 30 °C prior to western blot analysis (Fig. 7*A*). The HECTD1^HMW^ species that could be enriched for in the control reaction (Fig. 7*A*, lane 1) were readily processed by AnkOTU but not OTUB1∗ (Fig. 7*A*, lane 2 *versus* 3 and 1). We also probed these reactions with a monoclonal anti-ubiquitin antibody, which confirmed that the ubiquitin chains trapped by the TRABID catalytic dead constructs were also sensitive to TRABID DUB activity, but not to OTUB1∗ activity (Fig. 7*A*, lane 1 *versus* 2 and 3). This is in line with TRABID’s preference for binding and processing K29 and not K48 linkages.

To determine whether the observations made for GST-HECTD1^CD^ hold true in the context of full-length Hectd1, we next assessed the ubiquitin ligase activity of HA-tagged full-length mouse Hectd1. HA-FL mHectd1 was expressed and purified from HEK293ET and used in *in vitro* autoubiquitination assays (Fig. 7*B*). Indeed, we found that HA-full-length mHectd1^WT^ but not the catalytic dead mutant (C2587G) produced high-molecular-weight ubiquitinated species, which could be resolved following incubation with TRABID AnkOTU. In line with our previous data, Ub^K29R^ did not support full Hectd1 autoubiquitination activity. Together, this data indicates that the HECT domain of HECTD1, whether it be in isolation or in the context of the full-length protein, primarily assembles K29-linked ubiquitin chains, which are likely branched at K48.

### TRABID stabilizes HECTD1 levels

**Figure 8:**
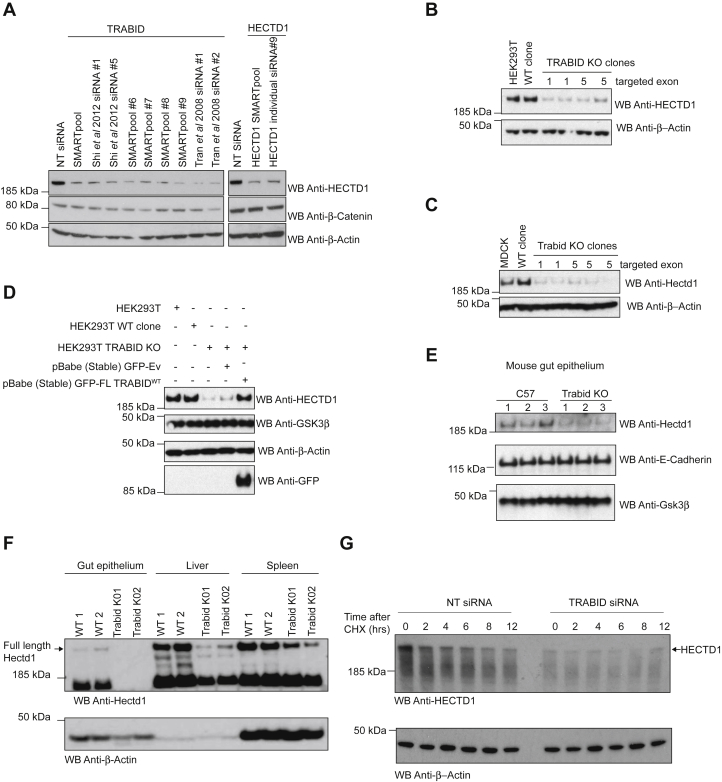
**TRABID stabilizes HECTD1 protein levels.***A*, HEK293ET were transiently transfected with 20 pmol of the indicated siRNA (TRABID SMARTpool siRNA, SMARTpool individual #6, #7, #8, #9, or individual siRNA from previous studies, as indicated) (32, 97). A nontargeting (NT) siRNA and HECTD1 siRNA were used as controls. Cell lysates were analyzed by western blot and probed for endogenous HECTD1. The housekeeping protein β-Actin and total β-Catenin were used as controls. CRISPR/Cas9 *TRABID* KO clones (exon 1 and 5 targeted) were analyzed for HECTD1 protein levels in HEK293T (*B*) and MDCK cells (*C*). *D*, loss of HECTD1 protein levels in HEK293T *TRABID* KO cells is rescued following the stable re-expression of pEGFP full-length TRABID, but not an empty pEGFP-Ev. GSK3β and β-Actin were used as loading controls. *E*, mouse gut epithelia were analyzed for Hectd1 protein levels in three *Trabid*^−/−^ or wild-type C57 mouse littermates. E-cadherin and Gsk3β were used as loading controls. *F*, the effect of *Trabid* depletion on Hectd1 levels was also observed in the liver and spleen tissues of those animals. *G*, cycloheximide chase in HEK293ET cells transfected with either a nontargeting siRNA or TRABID SMARTpool siRNA for 72 h followed by CHX addition (10 μg/ml). Samples were collected at the indicated time points post CHX addition and analyzed by western blot using HECTD1 and β-Actin antibodies.

Some E3 ligases have been shown to pair up with DUBs, which enables them to regulate each other’s activity and stability (40). Therefore, having established that HECTD1 is a novel E3 ligase assembling Ub^K29^ and Ub^K48^ chains, and a substrate of TRABID DUB activity, we next evaluated the functional relationship of this E3/DUB pair. Individual TRABID siRNAs were first validated for their ability to deplete pEGFP-FL TRABID (Fig. S7), prior to assessing their effect on endogenous HECTD1 protein levels in HEK293ET cells (Fig. 8). All TRABID siRNAs tested led to a decrease in endogenous HECTD1 protein levels (Fig. 8*A*). To further validate this, we recapitulated this data in HEK293T and MDCK CRISPR/Cas9 *TRABID* KO clones (Fig. 8, *B* and *C*, respectively). Importantly, this decrease in HECTD1 protein levels could be rescued by re-expression of pEGFP-TRABID^WT^ but not pEGFP-Empty vector alone, indicating that TRABID directly regulates HECTD1 levels (Fig. 8*D*). We extended these observations to *Trabid* KO mice (*Trabid*^−/−^), which showed a marked reduction in Hectd1 levels in the mouse gut epithelium, as well as liver and spleen tissues, compared with littermate controls (Fig. 8, *E* and *F*). Finally, we used a cycloheximide chase assay to study the steady-state level of HECTD1 upon TRABID depletion. This revealed that HECTD1 is readily turned over in the absence of TRABID, indicating that TRABID association with HECTD1 is required for its stability (Fig. 8*G*).

## Discussion

We previously established that the OTU deubiquitinase TRABID preferentially cleaves K29 and K33-linked diubiquitin (28). This, together with studies defining the biochemical properties of TRABID NZF 1, indicates that TRABID is highly tuned for the recognition and processing of these atypical linkages (29, 30, 31). Yet, the roles of TRABID DUB activity toward K29 and K33 linkages have remained elusive.

Proteomics studies of full-length TRABID wild type as well as of the catalytic dead trapping mutant TRABID^C443S^ have identified a number of candidate interactors and potential substrates including the E3 ubiquitin ligase HECTD1 (33, 39). Interestingly, most of the proposed mechanisms have been in the context of TRABID DUB activity toward K63-linked ubiquitin chains. Indeed, TRABID can clearly also process K63 linkages as shown *in vitro* on hexameric K63-linked ubiquitin chains and also in cells through the deubiquitination of APC (28, 33).

We first defined two catalytic dead TRABID constructs to identify putative TRABID substrates and also to determine the contribution of the OTU domain with regard to previously reported TRABID interactors. We reasoned that the overlap of candidate hits between TRABID^C443S^ and TRABID ΔOTU would implicate NZF interactors and therefore should identify likely substrates of TRABID DUB activity. In contrast, OTU-specific interactors (found in TRABID^C443S^ but not TRABID ΔOTU) might instead represent modulators/regulators of the OTU domain. The kinase/phosphatase STRIPAK complex was previously identified in a proteomics study of TRABID^WT^, and our data now show that this interaction is likely mediated by the OTU domain of TRABID (39, 70). This raises the possibility that despite its main role as a catalytic domain that mediates docking of ubiquitin chains for subsequent cleavage through a highly conserved catalytic triad, the OTU domain may have additional functions that are yet to be identified. As predicted, we were able to identify proteins (n = 50) common to the interactomes of both TRABID^C443S^ and TRABID ΔOTU. We hypothesized that this list would primarily include candidate TRABID substrates likely to be modified with K29- or K33-linked chains.

We validated HECTD1 as a direct substrate of TRABID DUB activity and showed that the NZF 1 of TRABID is the minimal domain that mediates binding to HECTD1. This implied that HECTD1 would be modified with either K29 and/or K33-linked chains in cells, given NZF 1’s affinity for these linkages (29, 31). This is interesting in light of previous studies identifying HECTD1 as a ligase assembling K63-linked polyubiquitin chains on APC and HSP90, in the context of Wnt signaling and cell secretion, respectively (32, 33, 71). HECTD1 has also been proposed to regulate K48-linked polyubiquitination on ACF7 in the context of EMT (72). Further, K48-linked polyubiquitin chains assembled by HECTD1 have also been implicated in the estrogen-dependent recruitment of transcriptional coactivator/corepressor (73). Therefore, we next aimed to reconcile TRABID’s preferred DUB activity toward K29 and K33 ubiquitin linkages, with the reported specificity of HECTD1 ligase activity for assembling K48 and K63-linked ubiquitin chains.

In contrast to RING E3 ligases, for which linkage specificity is dictated by E2-conjugating enzymes, the HECT domain plays a key role in determining the type of ubiquitin chain assembled. Therefore, we next determined HECTD1 ubiquitin ligase activity using *in vitro* autoubiquitination assays, UbiCREST, and Ubiquitin-AQUA. Biochemical activity assays showed that, *in vitro* at least, HECTD1 preferentially assembles K29 and K48-linked chains. Seminal work by the Pickart laboratory on HECT ligase specificity established that UBE3C can assemble mixed K29/K48 signals, and this has been further validated using TRABID NZF 1 to pull down ubiquitinated species from cells (29, 45, 65, 74, 75). Together, this led to the proposed model that K29-linked ubiquitin canonically exists as heterotypic and/or branched signals also containing K48-linkages. Although AIP4 (ITCH in human) and SMURF1 E3 ligases have been proposed to assemble K29 linkages on Deltex and Axin, respectively, the exact architecture of these chains remains unclear. This is also rather surprising given that NEDD4 family members predominantly generate K63-linked ubiquitin chains (76, 77).

Excitingly, our data shows that full HECTD1 ligase activity requires both K29 and K48 linkages, indicating the presence of branched linkages. Our findings also suggest that while HECTD1 and UBE3C can both use K29 and K48 linkages, the architecture of the chains assembled by either ligase is likely to be different, with HECTD1 being more reliant on branching compared with UBE3C. It will be important to combine recent approaches such as Ub middle-down MS/MS, UbProT, and Ub-clipping to further determine the architecture of these K29/K48 ubiquitin chains (78, 79, 80). For instance, middle-down MS/MS has been useful to quantify the abundance of branched and mixed linkages in cells while Ub-clipping has started to unravel ubiquitin chain architecture in the context of mitophagy (74, 80).

Branched chains were first proposed for K29-K48 in the context of the ubiquitin fusion degradation (UFD) pathway in yeast. Further studies revealed that K29-linked polyubiquitin chains assembled by Ufd4 are further modified with short K48 chains by the E4 enzyme Ufd2p (81, 82, 83). The yeast proteasome-associated ligase Hul5 (UBE3C in mammals) also acts as an E4 enzyme, extending ubiquitin chains on substrates bound to the proteasome (84). In the context of mammalian cells, UBE3C ligase activity has been implicated in the polyubiquitination of proteasomal receptor Rpn13 with K29/K48-linked polyubiquitin, which serves as a mechanism to decrease the recruitment of ubiquitinated substrates and decongest the 19S proteasomal lid during proteotoxic stress (66). Interestingly, heterotypic K29/K48-linked chains have also been found to modify cytosolic ERAD clients, with the ER-embedded E3 ligases TRC8 and UBE3C implicated in this mechanism (67). These studies, along with other reports on the function of branched K11/K48 and K48/K63 ubiquitin chains regulating mitosis and NFkB signaling respectively, have revealed yet another layer of complexity within the ubiquitin system (21, 22, 23). Our data now shows that although UBE3C and HECTD1 can assemble K29- and K48-linked chains, the exact architecture and regulation of these more complex ubiquitin chain types will need to be deciphered. In particular, it will be important to identify substrates of HECTD1 that are modified with these chains and also to determine the molecular functions of K29/K48 branched ubiquitin signals in cells. Together, our data identify TRABID-HECTD1 as the first DUB-E3 pair regulating K29-linked polyubiquitin chains. Interestingly, a recent study has shown that the proteasomal subunit RPN13 acts as an accessory protein to enhance the activity of the DUB UCH37 toward K48-containing, branched triubiquitin, and this could provide new ways to further explore the assembly/disassembly of these more complex ubiquitin chain types (85). The ability of some E3s to form branched ubiquitin chains, combined with this recent report of a debranching DUB activity, further exemplifies the versatility of protein ubiquitination.

Transient and genetic loss-of-function/rescue assays in mammalian cells showed that TRABID-HECTD1 is a functional DUB-E3 pair, with TRABID required to maintain HECTD1 stability. This is also true *in vivo* where Hectd1 protein levels were markedly decreased in tissues from *Trabid* KO mice. The list of validated DUB-E3 pairs is rapidly expanding and is providing new insights on how the stability or activity of these enzymes is regulated (86, 87). DUBs, for instance, have been shown to be particularly important for maintaining the stability of E3s, which can autoubiquitinate in the absence of a substrate, including BCA2, NEDD4L, WWP2, E6AP, Parkin, HRD1, and TRAF6 (88, 89, 90, 91, 92, 93, 94). For example, Hrd1 autoubiquitination has emerged as a key requirement for the retrotranslocation of some ERAD substrates, while TRAF6 autoubiquitination is a key event in NFκB signaling (93, 94).

Currently, none of the putative cellular functions of HECTD1 have been attributed to K29-linked polyubiquitination. Future interactome and substratome studies will be required to uncover the function of HECTD1 K29/K48 activity along with the molecular mechanisms involved. Interestingly, both the yeast (Ufd4) and *C. elegans* (Hecd1) ancestors of HECTD1 have been shown to function as UFD ligases (95). Our data is in line with these studies given that the UFD pathway implicates K29/K48 polyubiquitination. Therefore, it will be interesting to further explore the role of HECTD1 in the regulation of mutant ubiquitin UBB+1, which remains the best characterized physiological UFD substrate in human cells (96). Overall, our study conveys new insights into the regulation of atypical ubiquitin chains and also expands our understanding of DUB-E3 pairs.

## Experimental procedures

### Cell culture

COS-7, MDCK, HEK293T, and HEK293ET cells were grown in DMEM (Dulbecco’s modified Eagles Medium) supplemented with 10% fetal bovine serum (FBS) and 100 U/ml penicillin, 100 mg/ml streptomycin at 37 °C in 5% CO2. Cells were regularly checked for the presence of *Mycoplasma* using the MycoAlert *Mycoplasma* Detection Kit (Lonza Group AG).

### Transient siRNA knock down

TRABID siRNA used included ON TARGETplus SIRNA SMARTpool and individual siRNA #6, #7, #8 and #9 from Dharmacon (GE Healthcare, Dharmacon, Inc), individual siRNA for #1 and #2 (32), #1 and #5 (97). A nontargeting siRNA and ON TARGETplus SIRNA pool or an individual sequence for human HECTD1 were used as controls for HECTD1 levels. Oligos were synthesized by Eurofins Scientific (Luxembourg).

### Trabid (Zranb1) knockout mice

Animal care and procedures were performed in accordance with the standards set by the United Kingdom Home Office. Two ES cells clones were purchased from EUCOMM and injected into Blastocyst (TYR) and then injected into C57BL/6N (substrain JM8A1.N3) at the MRC Laboratory of Molecular Biology, Cambridge, in ES cells. To excise exon 3 and the neomycin cassette ZranG11 mice were crossed to P214 67deletor mice (98). To confirm the excision standard PCR was used with primers ZrnaGNeoF1: CTCATGCTGGAGTTCTTCGC; H5R2: CATACAAGCAAGCAAAAGATTCA. Genotyping was done by a standard PCR with primers H5F: GCTGTTCCAGTGGTCCTGAG; EX3R: TGGCTGCTAAGTCACCTTCC; LAR3: CACAACGGGTTC CTTCTGTTAGTCC.

### CRISPR/Cas9 genome editing in cells

*Trabid* KO cells were generated as described (99). Briefly, the design tool CRISPOR (crispor.tefor.net) was used to design single-stranded oligomers for sgRNA targeting vectors. RNA-encoding plasmid derivatives of pSpCas9(BB)2A-GFP (PX458) were generated by hybridizing single-stranded oligomers with their complementary strands in 2 mM Tris-HCl (pH 7.4), 10 mM NaCl, 200 mM EDTA at 95 °C for 5 min, and by subsequently ligating the double-stranded oligomers into a BbsI restriction site of PX458. HEK293T or MDCK cells were sorted 48 h posttransfection at a density of 1 cell/well in a 96-well plate and grown for 14 to 20 days to isolate individual clones. For genotyping, 2 to 5 × 10^4^ cells were homogenized in 15 μl MicroLYSIS-Plus (Microzone, Haywards Heath) and thermocycled as specified by the manufacturers. 2 μl of supernatant was used for PCR amplification, and the resulting PCR products were purified using the QIAquick purification kit (Qiagen, Hilden, Germany) and sequenced. Sequence chromatograms were analyzed using TIDE webtool (https://tide.deskgen.com/) and checked using MacVector software (MacVector Inc). Human gRNA and primers were used for HEK293T and dog gRNA and primers were used for MDCK cells:

Human *ZRANB1* Exon 1a (gRNA: CTAGAGTCTGGACATATCAA; Forward: GTGGCTTCCCGTTAATCTCA; Reverse: TCCAGTGCTGTGTCCTAG; Sequencing: CTTGAGCCAGATCCTGAG); human *ZRANB1* Exon 1b (gRNA: TCAGAGTCCCGCTTCGTAGC; Forward: GTGTCGTGCCCAAAGACCTA; Reverse: TACCTTTTCCCATCCCACGC; Sequencing: GTACCCAGTGCTTATCCC); human *ZRANB1* Exon 5 (gRNA: CTTGGAATTGGCTACACGTT; Forward: AACCTTGGTTCTCCGCTTCC; Reverse: AAACAGAAACCATGGACGTGT; Sequencing: GGTTCTCCGCTTCCTGTT).

Dog *Zranb1* Exon 1a (gRNA: GAGTCCCGTTTCATAGTAGG; Forward: TGGGATCCTTCTAGCACCGA; Reverse: ACGCATAGTGGGAGTACAGC; Sequencing: GTACCCAGTGCTTATCCC); dog *Zranb1* Exon 1b (gRNA: ATGAGCAAGACCGAGCTCGG; Forward: TGGGATCCTTCTAGCACCGA; Reverse: ACGCATAGTGGGAGTACAGC; Sequencing: GTACCCAGTGCTTATCCC); dog *Zranb1* Exon 5 (gRNA: TTTGGAATTGGCTACGCGTT; Forward: AGGCTTGGAACAGTTCAGTGT; Reverse: CCCCATAGCTTCCAAAGTAAAGG; Sequencing: GAGCCATAGATGTTTCAGC).

### Plasmids

pGEX-6p1 (GE Healthcare Life Sciences) encoding UBE3C, UBE2D1 (UBCH5A), UBE2D2 (UBCH5B), and UBE2D3 (UBCH5C) were contributed by Dr Thomas Mund (MRC Laboratory of Molecular Biology). Human HECTD1 catalytic domain (HECTD1^CD^) (AA2129-end) was amplified using cDNA from Normal Human Bronchio Epithelial cell line (NHBE) and cloned using BamH1/Xho1 into pGEX-6p1 (GST-HECTD1^CD^) or into pETM-11 using NCO1/Xho1 (His_6_-HECTD1) (46). Full-length HA-mouse-Hectd1 was a kind gift from Irene Zohn (71). Mouse full-length Hectd1 was subcloned into pEGFP-C1 (Clontech) and pCMV-3Tag1-3xFLAG (Agilent).

### Recombinant proteins

Expression and purification of TRABID Catalytic Domain (CD) (AnkOTU) have been described previously (28). His_6_-UBE1 (E-304), and DUBs including His_6_-Otubain-1 (OTUB1; E-522B), His6-OTUD7B/Cezanne (E-562), OTUD3_CD (E-574), AMSH/STABP (E-548B), USP2_CD (E-504), UCH37 (E-327), and USP14 (E-544) were purchased from R&D Systems, Inc. Bovine ubiquitin was purchased from Sigma-Aldrich-Aldrich (U6253). vOTU was gene synthesized (204AA, Mw = 23,241 g/mol) (56). AMSH∗ and OTUB1∗ have shown improved activity over AMSH and OTUB1, respectively. pOPINB-AMSH∗ was a gift from David Komander (Addgene plasmid # 66712; http://n2t.net/addgene:66712; RRID:Addgene_66712), pOPINB-OTUB1∗ was a gift from David Komander (Addgene plasmid # 65441; http://n2t.net/addgene:65441; RRID:Addgene_65441) (31).

Ubiquitin mutants including Ub^K0^, Ub^Met^, Ub^K/R^, and Ub^Konly^, and the recombinant human UbcH (E2) enzyme set protein (K-980B) were purchased from R&D Systems. GST-HECTD1 and GST-UBE3C, GST-tagged UBE2D1, 2, and 3 were expressed in *Escherichia coli* BL21 DE3 (RIL) (Agilent Technologies, Inc) and purified by affinity chromatography followed by gel filtration (GST-HECTs). GST-UBE2Ds were eluted with reduced glutathione prior to desalting with a HiTrap Desalting column. To remove the GST tag, GST-UBE2Ds were incubated overnight with PreScission protease (GE Healthcare) (28, 46). Fractions were concentrated, quantified using a NanoDrop 2000c, and 1 mg/ml stock was aliquoted, flash-frozen in liquid nitrogen, and stored at −80 °C. Ub^K29/K48only^ was expressed in BL21 DE3 (RIL), purified by perchloric acid extraction followed by ion exchange and gel filtration using established protocols, and the mass was check by liquid chromatography mass spectrometry using a 6545 LC/QTOF (Agilent Technologies) (100).

### Antibodies

Primary antibodies used were: anti-β-actin (Abcam Cambridge, 8227 or Sigma-Aldrich, A5441), anti-β-Catenin (Cell Signal, #9562), anti-E-Cadherin (Cell Signaling, #24E10), anti-HA High Affinity (Roche, 3F10), anti-FLAG M2 mouse monoclonal (Sigma-Aldrich, #A2220), anti-GST goat polyclonal (GE Healthcare, #27-4577-01), anti-GFP monoclonal antibody (GF28R) (Thermo Fisher Scientific, MA5-15256), anti-GSK3β (Cell Signaling, #27C10), anti-HECTD1 (Abcam, Ab101992), anti-HERC2 (Abcam, Ab85832), anti-UBE3C (Abcam, Ab180113), anti-TRIP12 (Abcam, Ab86220), anti-NEDD4 (Abcam, Ab14592), anti-polyubiquitin (linkage-specific K48 antibody [1001C]; Abcam, Ab190061), anti-ubiquitin rabbit polyclonal (MilliporeSigma-Aldrich; #07-375), anti-ubiquitin mouse monoclonal (Enzo Lifesciences; P4D1; BML-PW0930), and anti-His_6_-HRP (Sigma-Aldrich, #A7058).

Secondary antibodies used for ECL detection were goat anti-rabbit IgG-HRP (sc-2054), donkey anti-goat IgG-HRP (sc-2020), goat anti-mouse IgG-HRP (sc-2005), goat anti-rat IgG-HRP (sc-2032) (All from Santa Cruz Biotechnology, Inc). For near-infrared western blot detection, IRDye 680RD donkey anti-goat secondary (P/N 925-68074), IRDye 800CW goat anti-mouse secondary (P/N 925-32210), IRDye 680RD goat anti-mouse secondary (P/N 925-68070), and IRDye 680RD goat anti-rabbit secondary (P/N 925-68071) were used (All from LI-COR Biosciences).

### Transfection

Transfections of plasmid DNA in HEK293T cells were performed using either lipofectamine 2000 or polyethylenimine (PEI, linear, MW 25000, Polysciences). Typically, for 1 μg of plasmid DNA, 3 μl of a 1 mg/ml PEI solution was used. For siRNA, HEK293ET cells were transfected with lipofectamine 2000 as indicated by Thermo Fisher Scientific.

### Immunoblotting

Cells were lysed in Triton Lysis Buffer (150 mM NaCl, 25 mM Tris pH 7,4, 1% Triton X-100 supplemented with cOmplete EDTA-free protease inhibitor cocktail) or RIPA (150 mM NaCl, 50 mM Tris pH 8, 1% NP40, 0.5% NaDoc, 0.1% SDS, supplemented with cOmplete EDTA-free protease inhibitor cocktail) as indicated. Cell lysates were denatured in 4X LDS/100 mM DTT and resolved on 4 to 12% Bris-Tris or 3 to 8% Tris Acetate SDS-PAGE gels under reducing conditions and transferred to a polyvinylidene difluoride membrane (PVDF, 0.45 μM, Thermo Fisher Scientific) or Millipore Immobilon FL for near-infrared fluorescence detection by LI-COR Clx. Membranes were blocked in 5% (w/v) nonfat dried skimmed milk powder in PBST (PBS, 0.1% Tween-20) for 1 h at room temperature (RT). Membranes were then probed with the appropriate primary antibodies in blocking buffer overnight at 4 °C. Detection was performed by incubating membranes with the appropriate horseradish peroxidase (HRP)-conjugated or IRDye secondary antibodies in blocking buffer at RT for 1 h. Enhanced chemiluminescence (ECL) (Thermo Fisher Scientific) was used for anti-ubiquitin western blots, and images were acquired on a FUSION-SL imager (Vilber Lourmat, France). Alternatively, anti-GST blots were visualized on a LI-COR Clx (46).

### Western blot for mouse tissue analysis

For mice tissues analyses, small intestines were removed immediately after cervical dislocation, opened longitudinally, and washed with cold PBS. Remaining mucosa was removed with Kimcare tissue (Kimberly-Clark). The epithelia were separated from their underlying mucosa by scraping the small intestines with a razor blade, flash-frozen in liquid nitrogen, and stored at −80 °C for less than 24 months. Protein extracts were prepared from mouse small intestine epithelium by adding a small fragment of flash-frozen mouse gut directly into NuPage LDS loading buffer containing 5% β-mercaptoethanol and mechanically homogenizing on ice. Extracts were sonicated twice for 10 s on ice before western-blot analysis.

### Immunoprecipitation

Cells were transfected with 500 ng of each plasmid per well of a 6-well plate using Lipofectamine 2000 (Thermo Fisher Scientific) in Opti-MEM using a 3:1 Lipofectamine 2000:DNA ratio for 24 h to 48 h. Each well was lysed in 400 μl of Triton Lysis Buffer for 15 min on ice. Lysates were cleared by centrifugation at 13,000 rpm for 15 min at 4 °C. FLAG-tagged proteins were captured using FLAG M2 magnetic beads (Sigma-Aldrich) while GFP-tagged proteins were captured with GFP-Trap_MA (ChromoTek GmbH, Germany) for 1 h at RT, as per the manufacturer’s protocol. Beads were washed four times in Triton Lysis Buffer and denatured at 95 °C for 5 min in 2X LDS/100 mM DTT. Samples were then resolved on 4 to 12% Bis-Tris or 3 to 8% Tris Acetate SDS PAGE gels and analyzed by western blotting. For immunoprecipitation of endogenous HECT ligases, 2 μg of Dynabeads magnetic beads protein G (Thermo Fisher Scientific, #10003D) was washed three times in Triton Lysis Buffer prior to coupling with 4 μg of anti-HECT antibody. Following 1 h incubation at RT on a rotating wheel, anti-HECT-coupled magnetic beads were washed three times in Triton Lysis Buffer and added to 390 μl of lysis buffer, while 10 μl of each lysed sample was kept as input controls. Following 1 h incubation at RT, beads were washed four times in Triton Lysis Buffer, and both the input and IP samples were denatured at 95 °C for 5 min in 2X LDS/100 mM DTT. Samples were then resolved on 4 to 12% SDS PAGE gels and analyzed by western blotting. Typically, the input ran on a gel represented 2.5% of the initial 400 μl lysate, and we ran half of the final denatured IP samples. HA-tagged full-length HECTD1 was expressed and purified from HEK293ET using Pierce^TM^ anti-HA Magnetic Beads (Thermo Fisher Scientific, #88836) using standard procedures (28).

For ubiquitin trapping IP (Fig. S1*B*), 3xFLAG TRABID constructs (500 ng/well of a 6-well plate) were transfected with HA-ubiquitin (500 ng/well of a 6-well plate) with lipofectamine 2000 in HEK293ET cells. Twenty-four hours post transfection, cells were harvested, lysed in Triton Lysis Buffer, and FLAG-tagged proteins were captured using FLAG M2 magnetic beads (Sigma-Aldrich). Input and IP samples were analyzed by western blotting using anti-FLAG and anti-HA antibodies, respectively.

To further validate TRABID-HECTD1 interaction, 250 ng of pEGFP-tagged and 3xFLAG-tagged TRABID and Hectd1 plasmids were co-expressed for 24 h in 6-well plates, in antibiotic-free DMEM/FBS. PEI was used for transfection. Twenty-four hours post transfection, cells well rinsed with PBS, lysed in Triton Lysis Buffer, and immunoprecipitation assays were carried out as mentioned above.

### Cycloheximide (CHX) chase

HEK293ET cells were transiently transfected with 20 pmol of SMARTpool siRNA for 72 h prior to incubation with 10 μg/ml of cycloheximide. Samples were collected at the indicated time points and lysed in RIPA (supplemented with 1X EDTA-free protease inhibitor tablets).

### *In vitro* autoubiquitination assay

Autoubiquitination assays were carried out in 10 μl reactions containing 100 ng of His_6_-E1, 500 ng of indicated E2, 2.5 μg of His or GST-tagged HECT E3 ubiquitin ligase, 2.5 μg of ubiquitin, in 1× ubiquitination assay buffer (25 mM Tris, pH 7.4, 20 mM NaCl, 10 mM MgCl_2_, 1 mM DTT, 1 mM ATP) (46). Reactions were carried out at 30 °C for 3 h and stopped by addition of 2X LDS Sample Buffer/100 mM DTT. Samples were then analyzed by immunoblotting.

### Ubiquitin chain restriction analysis (UbiCREST)

UbiCREST was carried out as described (55, 56). Briefly, samples obtained from *in vitro* autoubiquitination were treated for 20 min at RT with 2 mU of Apyrase (Sigma-Aldrich, A2230) prior to incubation 1X DUB buffer (50 mM NaCl, 50 mM Tris, pH 7.4 and 50 mM DTT) with TRABID AnkOTU (1 or 2.5 μM, as indicated), OTUB1/OTUB1∗ (2 μM), OTUD7B/Cezanne (1 μM), AMSH/AMSH∗ (2 μM), OTUD3_CD (2 μM), USP2_CD (1 μM), yUbp6 (1 μM), vOTU (3 μM), UCH37 (200 nM), USP14 (1 μM). Reactions were arrested by addition of 2X LDS/100 mM prior to western blot analysis using the indicated antibodies.

### UbiCREST assay of trapped endogenous polyubiquitinated HECTD1 (HECTD1^HMW^)

Five micrograms of 3xFLAG-TRABID FL TRABID^C443S^ was transiently transfected in one 10 cm dish of HEK293T cells using PEI. Twenty-four hours post transfection, cells were rinsed with PBS and lysed Triton Lysis Buffer (supplemented with EDTA-free 1X protease inhibitor). Twenty microliters of Dynabeads protein G magnetic beads slurry was rinsed with lysis buffer and incubated for 2 h at RT with 4 μg of HECTD1 antibody, as per manufacturer’s recommendations. Following coupling, beads were rinsed three times in lysis buffer and incubated with the lysate from 3xFLAG-TRABID FL TRABID^C443S^-expressing HEK293T cells. Two hours after incubation, beads were captured using a magnet and washed four times in lysis buffer and twice in 1X DUB buffer. Beads were finally incubated in a 10 μl reaction and incubated with AnkOTU (1 μM) or OTUB1∗ (2 μM). A control reaction without DUB treatment was also carried out. Samples were incubated for 1 h at 30 °C upon which reactions were arrested by addition of 2X LDS/100 mM DTT, resolved on a 3 to 8% PAGE gel, and analyzed by western blotting.

### GST pull-down

Autoubiquitination assays obtained with Ub^K0^, Ub^WT^, or Ub^K29R^ and subjected to UbiCREST were used in GST pull-down experiments. GST pull-down was carried out as described previously (26). Briefly 20 μg of GST or GST-TRABID NZF 1 to 3 (AA1-200) was incubated with 20 μl of Glutathione Magnetic Agarose Beads (Thermo Fisher Scientific) for 1 h at RT in 500 μl of pull-down buffer (PDB; 150 mM NaCl, 25 mM Tris pH 7.4, 5 mM DTT and 0.1% NP-40), and then washed four times with PDB. Washed beads were incubated with each of the indicated *in vitro* autoubiquitination assays in 500 μl of PDB supplemented with BSA (0.5 mg/ml) overnight at 4 °C. Beads were washed five times in PDB, and following the final wash beads were mixed with 2X LDS/100 mM DTT. Samples were analyzed on a 4 to 12% SDS PAGE followed by western blotting using anti-ubiquitin mouse monoclonal (Enzo Lifesciences; P4D1) or by silver staining (ProteoSilver, Sigma-Aldrich).

GST pull-down was also used to further validate the interaction between TRABID and HECTD1. Here 20 μg of GST, GST-TRABID NZF 1 to 3, or GST-TRABID NZF 1 to 3^TY>LV^ was coupled to Glutathione Magnetic Agarose Beads for 1 h at RT followed by washes with PDB. One 10 cm dish of HEK293T cells was lysed in Triton Lysis Buffer supplemented with EDTA-free protease inhibitor tablet (1X) for 20 min on ice followed by centrifugation at 15,000 rpm for 15 min in a refrigerated centrifuge. The cleared lysate was split into three equal parts and incubated with the GST-coupled beads for 2 h at RT in PDB supplemented with BSA (0.5 mg/ml). Beads were washed five times in PDB and finally mixed with 2X LDS/100 mM DTT prior to western blotting.

### Interactome studies

#### Mass spectrometry

Twenty 175 cm^2^ flasks of subconfluent HEK293ET cells were transfected with pCMV-3xFLAG_EV, pCMV-3xFLAG_Full-length human TRABID^C443S^ or pCMV-3xFLAG_TRABID 1-339/(=ΔOTU). Cells were lysed in Triton Lysis Buffer and cleared by centrifugation for 20 min at 13,000 rpm at 4 °C. Supernatant was incubated for 1 h at room temperature with FLAG M2 magnetic beads (Sigma-Aldrich) followed by four washes with 1 ml of lysis buffer. Beads were finally resuspended in 2X LDS sample buffer/100 mM DTT and incubated at 95 °C for 5 min. Denatured samples were then resolved on a 4 to 12% Bis-Tris SDS polyacrylamide gel, and the gel was stained with Imperial Protein Stain (Thermo Fisher Scientific). IP experiments were n = 1 for each of the three plasmids.

Polyacrylamide gel slices (1–2 mm) containing the purified proteins were prepared for mass spectrometric analysis by manual *in situ* enzymatic digestion. Briefly, the excised protein gel pieces were placed in a well of a 96-well microtiter plate and destained with 50% v/v acetonitrile and 50 mM ammonium bicarbonate, reduced with 10 mM DTT, and alkylated with 55 mM iodoacetamide. After alkylation, proteins were digested with 6 ng/μl Trypsin (Promega) overnight at 37 °C. The resulting peptides were extracted in 2% v/v formic acid, 2% v/v acetonitrile. The digest was analyzed by nanoscale capillary LC-MS/MS using a Ultimate U3000 HPLC (ThermoScientific Dionex) to deliver a flow of approximately 300 nl/min. A C18 Acclaim PepMap100 5 μm, 100 μm × 20 mm nanoViper (ThermoScientific Dionex), trapped the peptides prior to separation on a C18 Acclaim PepMap100 3 μm, 75 μm × 250 mm nanoViper (ThermoScientific Dionex). Peptides were eluted with a gradient of acetonitrile. The analytical column outlet was directly interfaced *via* a nanoflow electrospray ionization source, with a hybrid dual pressure linear ion trap mass spectrometer (Orbitrap Velos, ThermoScientific). Data-dependent analysis was carried out, using a resolution of 30,000 for the full MS spectrum, followed by ten MS/MS spectra in the linear ion trap. MS spectra were collected over an m/z range of 300to 2000. MS/MS scans were collected using a threshold energy of 35 for collision-induced dissociation. LC-MS/MS data were then searched against a protein database (UniProt KB, 2019. Swiss-Prot, 563,552 entries. TrEMBL, 195,104,019 entries) using the Mascot search engine program, version 2.4 (Matrix Science) (101). Database search parameters were set with a precursor tolerance of 5 ppm and a fragment ion mass tolerance of 0.8 Da. Trypsin specificity was set as C-terminal side of lysine and arginine amino acid unless a proline residue was present on the carboxyl side of the cleavage site. Two missed enzyme cleavages were allowed, and variable modifications for oxidized methionine, carbamidomethyl cysteine, pyroglutamic acid, phosphorylated serine, threonine and tyrosine, along with GlyGly and LeuArgGlyGly lysine were included. MS/MS data were validated using the Scaffold program (Proteome Software Inc) (102) with a 0.2% FDR calculated with peptide probabilities estimated using ProteinProphet. All data were additionally interrogated manually.

#### Data interrogation

From an initial list of 2225 proteins (95% protein threshold, minimum of 2 unique peptides in any of the three conditions), proteins with one or more unique peptide in the control IP (3xFLAG-Empty vector) were removed, resulting in a working list of 208 proteins. We also included in this list seven proteins that did not fulfil the criterion above, but which were nevertheless included on the basis that these were part of either the STRIPAK complex (STRN, STRN3, Zinedin) or E3 ligases (HERC2, MYCBP2, UBR4, UBE3A) (Table S1). From this list of 215 proteins, we then applied criteria of zero unique peptide in the TRABID ΔOTU condition and at least two unique peptides in the FL TRABID^C443S^ condition, which yielded 103 OTU-specific candidates. We applied criteria of zero unique peptide in the TRABID FL^C443S^ condition and at least two unique peptides in the TRABID ΔOTU condition, which produced 23 TRABID ΔOTU-specific candidates. Finally, we applied criteria of zero unique peptide in the empty vector control IP and at least two unique peptides in TRABID FL^C443S^ and/or TRABID ΔOTU, which resulted in 50 candidate substrates.

### Ubiquitin-absolute QUAntification (ubiquitin-AQUA) analysis

GST-HECTD1^CD^ was used in autoubiquitination assay to generate polyubiquitinated GST-HECTD1^CD^ using either ubiquitin WT (Ub^WT^) or Ub^K29R^ (R&D Systems). Reactions were stopped after 3 h with addition of 2X LDS/100 mM DTT. In order to analyze the ubiquitin chains left on GST-HECTD1^CD^ following cleavage of K29-linked ubiquitin chains, autoubiquitination reaction with GST-HECTD1^CD^ was arrested after 3 h using 2 mU of Apyrase for 20 min at RT prior to incubation with 2.5 μM of TRABID AnkOTU. Five microliters of each reaction was resolved on a 4 to 12% Bis-Tris PAGE gel and detected by western blot using an anti-ubiquitin antibody. The remaining 30 μl was run on a 4 to 12% Bis-Tris PAGE gel stained with Coomassie stain. Gel slices, two areas per experimental condition (A/B/C1, A/B/C2), corresponding to polyubiquitinated GST-HECTD1^CD^ were cut out of the gel (Fig. S4*B*), subjected to in-solution trypsin digestion, and quantitatively analyzed by LC-MS/MS as reported (57, 103, 104, 105). A heavy labeled peptide standard mix representing all seven Lys-linkages (K6, K11, K27, K29, K33, K38, and K63; Thermo Fisher Scientific/Sigma-Aldrich, Table S2) was prepared and spiked into the digested peptide samples at a final concentration of 10 fmol/μl) prior to LC-MS/MS analysis in data-dependent analysis (DDA) mode. For MS data analysis and quantification, a targeted proteomics protocol adapted to MS/MS acquisition was used based on the Skyline workflow and adapted for measuring the absolute quantity of Ub-linkages (Ub-AQUA) (106). To this end, raw MS files were processed through ProteoWizard to generate mgf files that were searched using the Mascot search engine as described above. Mascot data/files were used and imported into the Skyline software (version 64 bit, 19.1.0.193) to generate a ubiquitin-specific peptide library including all the Ub linkages represented as Lys-GlyGly modifications as well as heavy labeled standards. Raw MS files were then imported into the Skyline software and matched against the Ub-peptide library. Single-ion chromatograms were extracted automatically, representing the m/z of Ub derived tryptic peptides carrying Lys-GlyGly modifications. To calculate the Ub-linkage abundance, the heavy (standard) *versus* light (experiment) abundance ratios determined based on the MS1 peak intensities (Fig. S4*A* and Table S2).

### Microscopy

COS-7 cells were transfected with the indicated pEGFP-tagged TRABID plasmids (Fig. S1*C*) fixed and visualized as previously described (28). For Fig. S7, HEK293ET cells were transfected with pEGFP-full-length TRABID^WT^ (200 ng/well of a 24-well plate) together with 20 pmol of the indicated siRNA. Forty-eight hours following transfection, cells were fixed with 4% paraformaldehyde and imaged on an EVOS Cell Imaging System (Thermo Fisher Scientific).

## Data availability

The mass spectrometry proteomics data have been deposited to the ProteomeXchange Consortium *via* the PRIDE partner repository with the data set identifier PXD022703 (107).

## Conflict of interest

The authors declare that they have no conflicts of interest with the contents of this article.
