# Supplementary material for: The deubiquitinase TRABID stabilizes the K29/K48-specific E3 ubiquitin ligase HECTD1
Source: J Biol Chem. 2021 Jan 8;296:100246. doi: 10.1074/jbc.RA120.015162 (PMC7948964; doi:10.1074/jbc.RA120.015162)
Supplement: Supplemental Figure and Tables [file mmc1.pdf]

Supporting information

Supplementary Fig 1

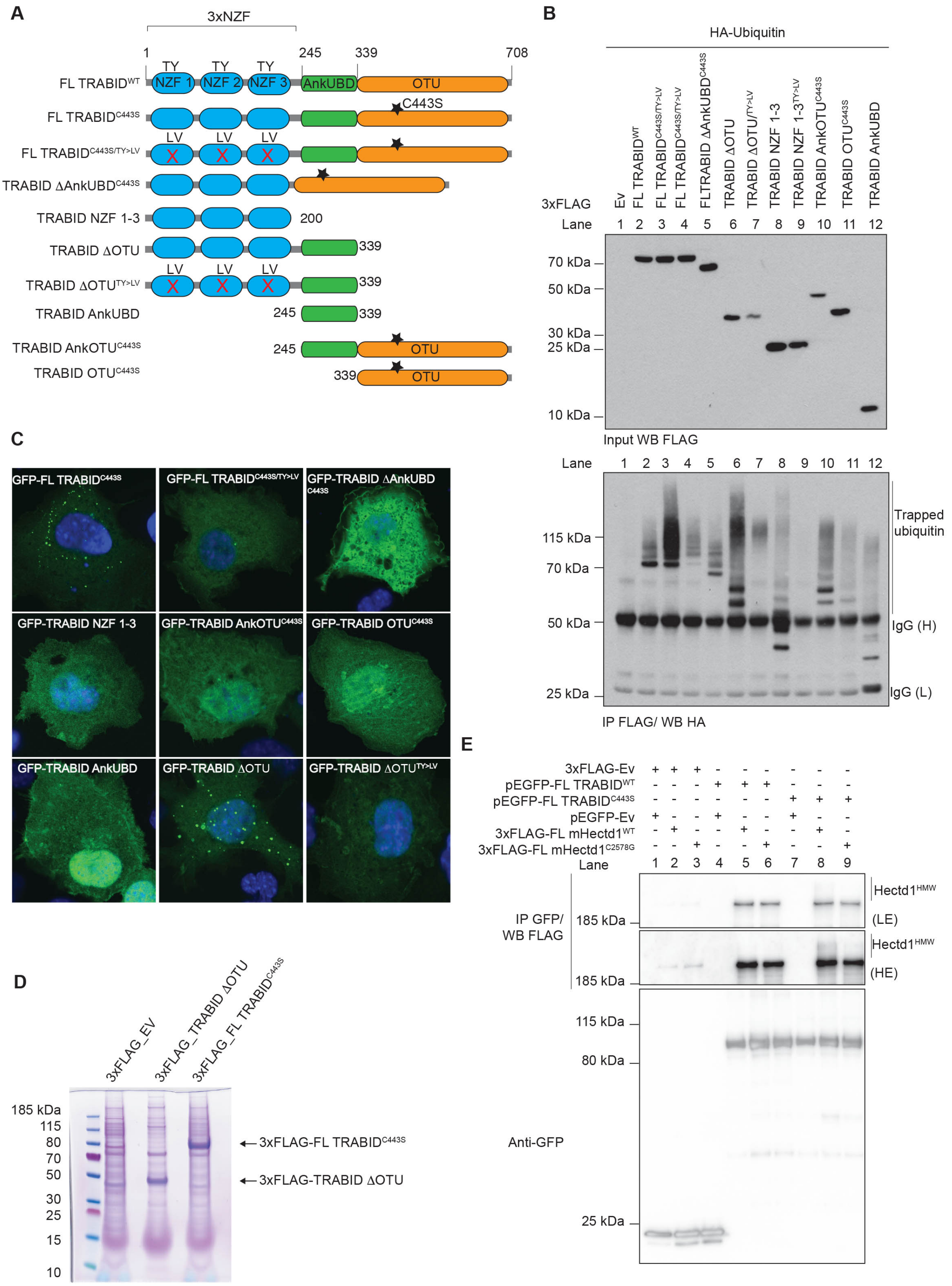

Supplementary Figure 1. TRABID catalytic dead traps polyubiquitinated species.

A) Domain organisation of TRABID constructs. NZF TY>LV mutants abrogates the ubiquitin binding property of NZF domains (Red cross, LV); C443S abrogates DUB activity (Black star) (1). **B)** Immunoprecipitation assays showing that catalytic dead TRABID constructs require functional NZFs for trapping ubiquitin species. The indicated pCMV-3xFLAG_TRABID constructs and HA-Ubiquitin

were expressed for 24hrs in HEK293ET cells. Cell lysates were subjected to immunoprecipitation using FLAG® M2 magnetic beads. Input samples were probed with FLAG, and FLAG IP samples were probed with anti-HA. Note that TRABID NZF 1-3 was less efficient at trapping ubiquitin compared to FL TRABID^{C443S} or TRABID ΔOTU (Lane 8 vs. 3 & 6). **C)** Loss of TRABID DUB activity results in the formation of puncta in cells (2), and this is dependent on the ubiquitin binding property of the NZF domains. **D)** Coomassie gel showing the expression of pCMV-3xFLAG_Ev, catalytic dead constructs pCMV-3xFLAG_TRABID ΔOTU and pCMV-3xFLAG_FLTRABID^{C443S} used for the proteomics experiments (Fig 1). **E)** Immunoprecipitation assays showing the interaction between ectopically expressed TRABID and Hectd1. pEGFP_Ev, pEGFP _FL TRABID^{WT} or pEGFP_FL TRABID^{C443S} were transiently co-expressed with pCMV-3xFLAG_Ev, pCMV-3xFLAG_FL mouse Hectd1^{WT} or pCMV-3xFLAG_FL mouse Hectd1^{C2587G} as indicated. GFP-TRAP beads (Chromotek) were used to immunoprecipitate GFP-tagged proteins. Note the enrichment of higher molecular weight species of Hectd1 (Hectd1^{HMW}) when co-expressed with catalytic dead TRABID (IP GFP/WB Anti-FLAG, lane 8 vs. 5). Hectd1^{HMW} species are markedly reduced upon co-expression with catalytic dead Hectd1 (IP GFP/WB Anti-FLAG, lane 8 vs. 9). This indicates that TRABID catalytic dead can therefore trap Hectd1^{HMW} species which appear to represent autoubiquitinated Hectd1. High exposure images are shown to help visualisation of Hectd1^{HMW}.

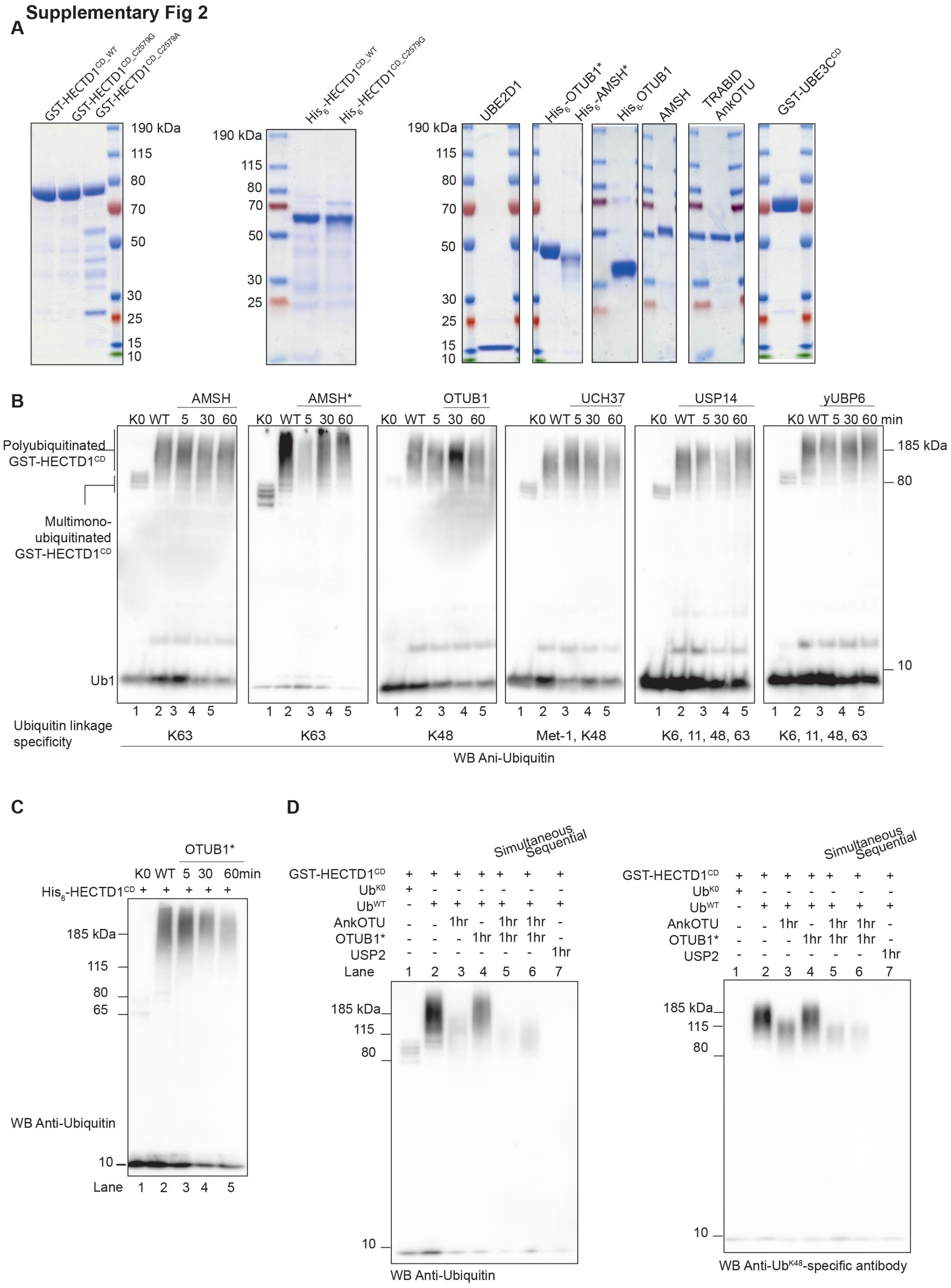

Supplementary Figure 2. Analysis of HECTD1 ligase activity by UbiCREST and Ub-AQUA.

A) Coomassie stained gels showing recombinant proteins made in-house and used for autoubiquitination and UbiCREST assays (5-15 µg loaded). **B)** Time-course UbiCREST assays were performed using autoubiquitinated GST-HECTD1^{CD} as substrate, as in Figure 5. Briefly, autoubiquitination assays were performed using ATP, UBE1, UBE2D1 and GST-HECTD1^{CD}. Following 3 hrs at 30ºC, reactions were terminated through the addition of 2 mU apyrase for 20

minutes prior to addition of the K63-specific AMSH (2 μM), AMSH* (2 μM) (3), OTUB1 (2 μM) or the proteasomal DUBs UCH37 (200 nM) (4,5) and USP14/yUbp6 (1 μM) (6,7). Note that AMSH* and OTUB1* have improved activity over AMSH and OTUB1, respectively (3). UbiCREST reactions were terminated at the indicated times by addition of 2X LDS/100 mM DTT, resolved on 4-12% Bis-Tris SDS PAGE gel and analysed by western blotting using an anti-ubiquitin antibody (P4D1). **C)** UbiCREST was performed using OTUB1* (2 μM) as in Fig 5A but using His_6-HECTD1^{CD}. **D)** Double UbiCREST assay was performed as in Fig 5B but using GST-HECTD1^{CD}. Note that similar data were obtained whether GST-tagged or His_6-tagged HECTD1^{CD} was used. Reactions were probed with a monoclonal anti-Ubiquitin antibody (P4D1) or an anti-Ub^{K48}-specific antibody, as indicated.

Supplementary Fig 3

A

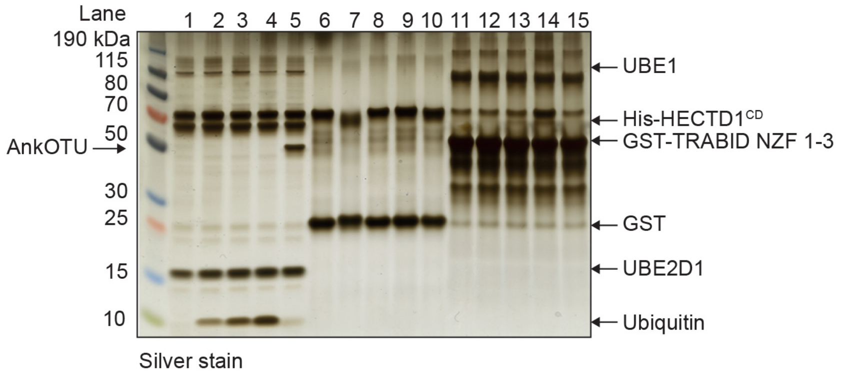

Supplementary Figure 3. Loading of proteins used for the GST pulldown in Figure 5D.

Protein loading control for the GST pulldown experiment shown in Fig 5D.

Supplementary Fig 4A

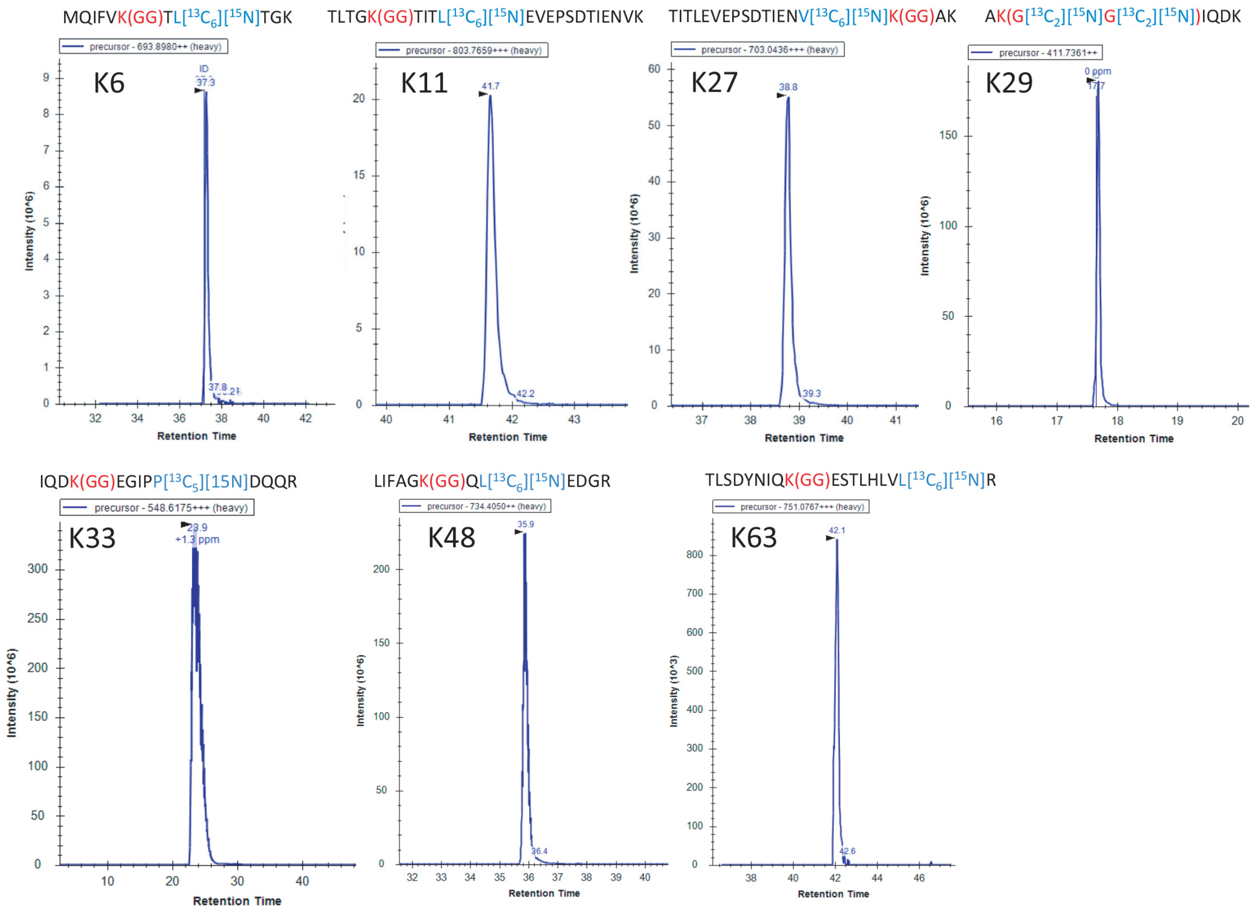

Supplementary Fig 4B-D

B

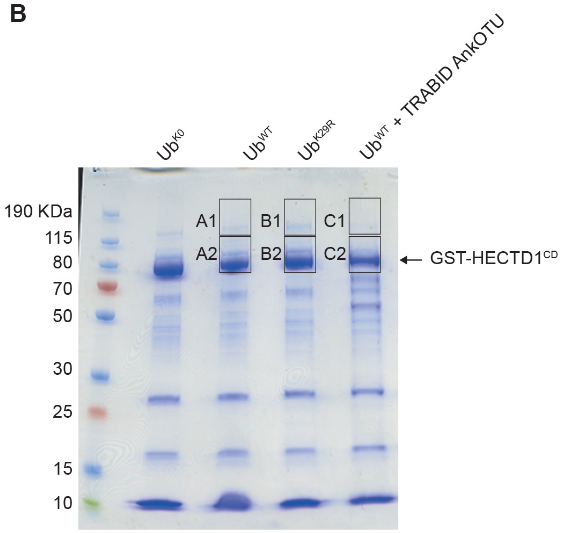

C

%	A1	A2	A overall	B1	B2	B overall	C1	C2	C overall
**K6**	1.23%	9.06	5.14	9.25	84.10	46.68	15.97	3.26	9.61
**K11**	5.02	7.03	6.03	10.90	0.51	5.70	22.93	15.83	19.38
**K27**	0.12	0.01	0.07	0.41	0.00	0.21	0.22	0.09	0.15
**K29**	30.81	24.54	27.67	0.60	0.01	0.31	1.06	0.62	0.84
**K33**	7.96	0.14	4.05	1.00	0.05	0.53	2.04	1.53	1.79
**K48**	37.76	50.15	43.96	50.46	9.44	29.95	54.94	68.67	61.80
**K63**	17.10	9.07	13.08	27.38	5.87	16.63	2.83	10.01	6.42
	Ub^{WT}			Ub^{K29R}			Ub^{WT} + TRABID AnkOTU		

D

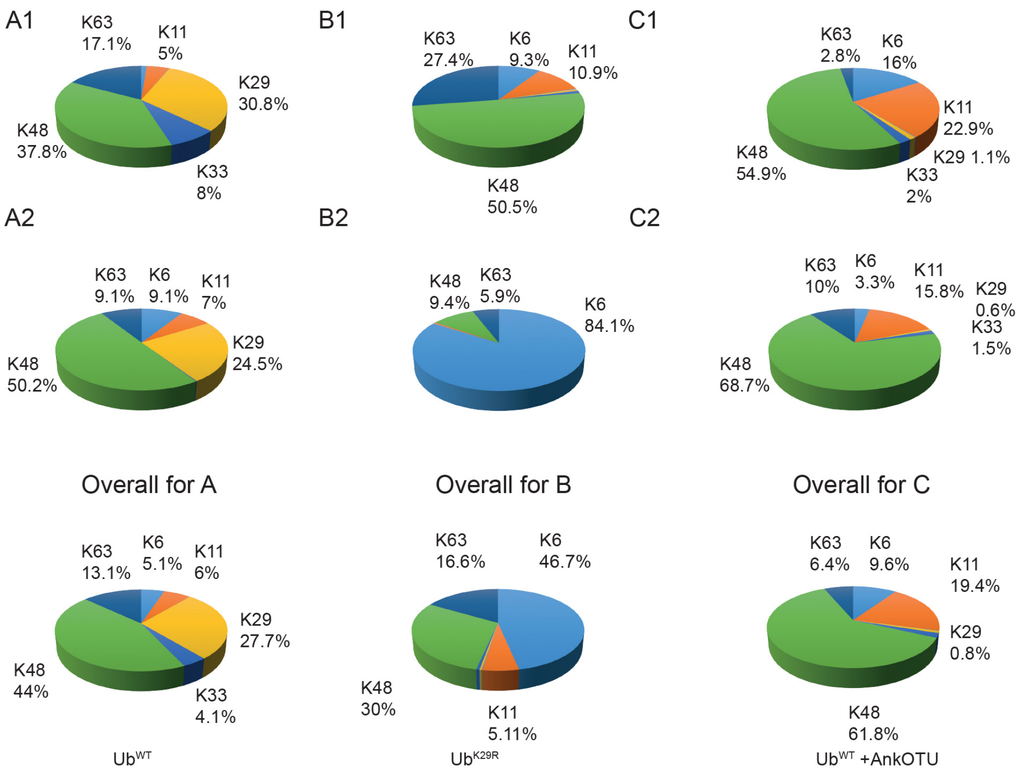

Supplementary Figure 4. Analysis of HECTD1 and UBE3C ligase activity by UbiCREST.

A) Skyline data showing single ion chromatograms representing the m/z of Ub derived tryptic peptides carrying Lys-GlyGly modifications. **B)** Coomassie stained gel showing the *in vitro* autoubiquitination reactions using ubiquitin wild type (Ub^{WT}), Ub^{K29R} and Ub^{WT} followed by UbiCREST with TRABID AnkOTU, which were analysed by ubiquitin-AQUA. Two gel slices were collected for each lane and analysed, as depicted (boxed areas on gel). **C)** Table showing the percentage of each ubiquitin linkage as detected by ubiquitin-AQUA, for each gel section and also for the entire lane (See Supplementary Table 2 for additional raw data). **D)** Pie chart showing ubiquitin linkage abundance (Overall data were shown in Fig 5F-H).

Supplementary Fig 5

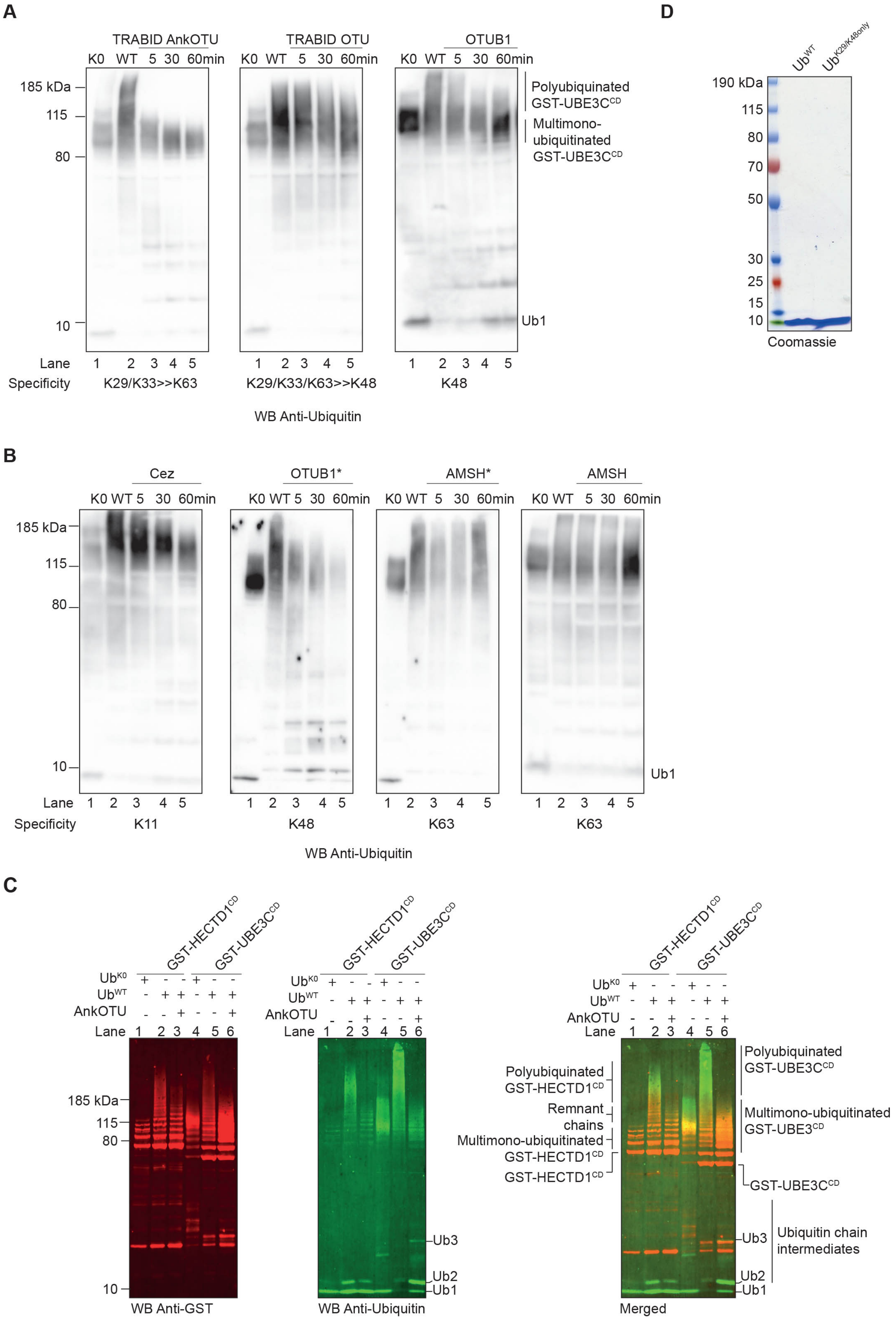

Supplementary Figure 5. UbiCREST assays for GST-UBE3C^{CD}.

Time-course UbiCREST assays were performed using autoubiquitinated GST-UBE3C^{CD} as substrate using **A)** TRABID AnkOTU (2.5 µM), TRABID OTU (2.5 µM), OTUB1 (2 µM) and **B)** Cezanne (1 µM), OTUB1* (2 µM), AMSH (2 µM) and AMSH* (2 µM). **C)** LI-COR detection of UbiCREST assays performed as in Fig 6A using GST-HECTD1^{CD} (Lanes 1-3) and GST-UBE3C^{CD} (Lanes 4-6). HECTD1 and UBE3C are the only two HECT ligases shown to assemble Ub^{K29} and Ub^{K48}-linked chains (3,8,9). Following incubation for 3 hrs at 30ºC, reactions were terminated through the addition of 2 mU apyrase for 20 minutes prior to addition of TRABID AnkOTU (2.5 µM). Reactions were analysed by western blotting using a mouse anti-ubiquitin (Enzo Lifesciences; P4D1) and an Anti-GST antibody followed by incubation with LI-COR IRDye® secondary antibodies and detection on a LICOR Odyssey CLx. Note the production of free ubiquitin chains (Ub2, 3 etc) in the GST-UBE3C^{CD} reaction treated with AnkOTU (Lane 6 vs. 3). Short remnant chains can be detected for GST-HECTD1^{CD} treated with AnkOTU (Lane 3 vs. 1). **D)** Coomassie stained gel showing purified recombinant Ub^{WT} and Ub$^{K29/K48only}$ (10 µg loaded).

Supplementary Figure 6

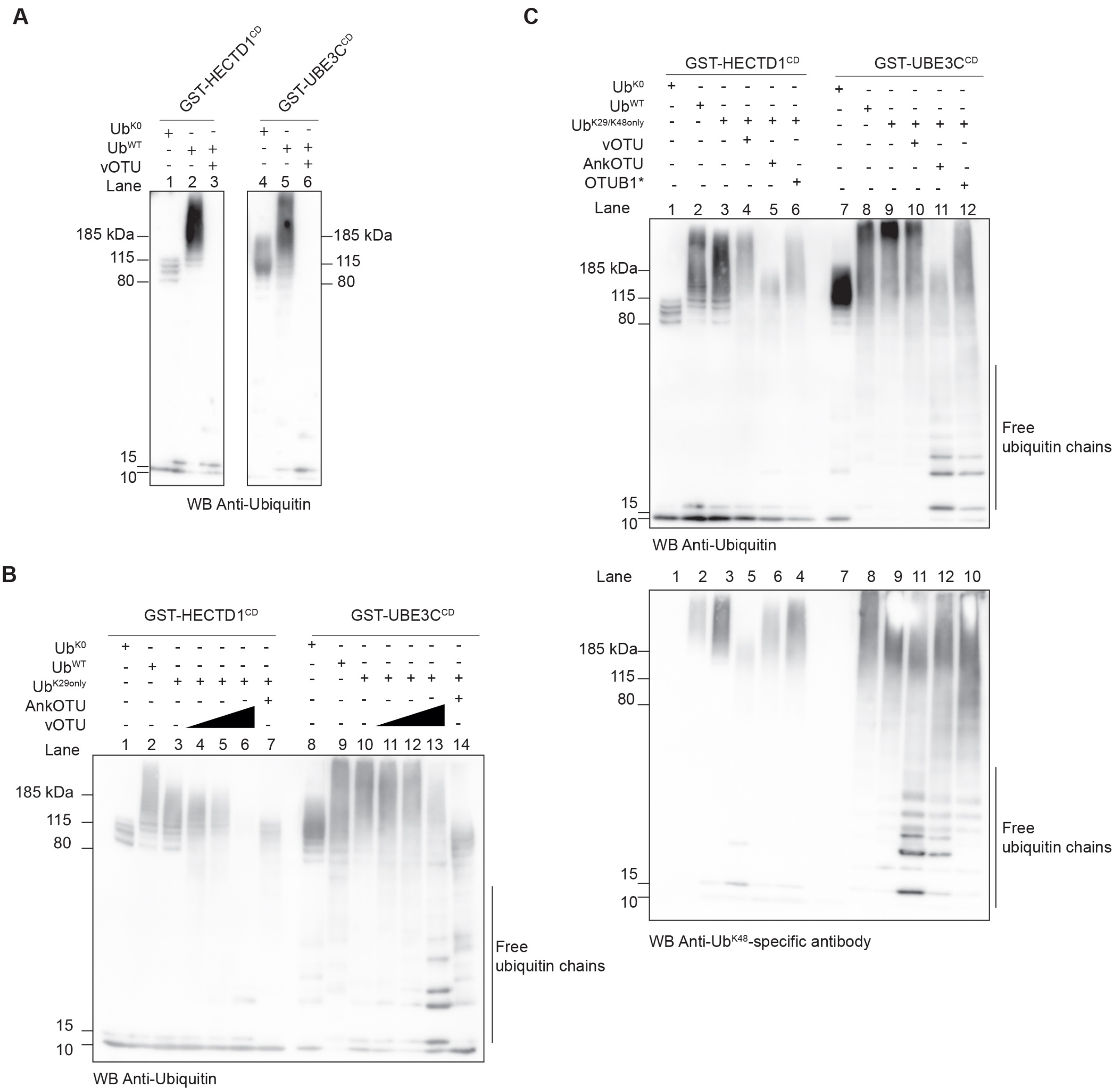

Supplementary Figure 6. UbiCREST analysis using vOTU.

A) UbiCREST assays were performed as previously described using vOTU for 1 hr (3 μM = 0.069 ng/μl). Note the complete disappearance of the ubiquitinated signals in lanes 3 and 6. **B)** vOTU concentration was titrated to try and determine a concentration which would not lead to the processing of K29only-linked chains. Increasing concentrations of vOTU were used: 0.86 μM (0.02 ng/ul) (Lanes 4 and 11), 8.6 μM (0.2 ng/μl) (Lanes 5 and 12), 86 μM (2 ng/μl) (Lanes 6 and 13) (10). **C**) UbiCREST assays were performed using Ub$^{K29/K48only}$ to generate polyubiquitinated HECT ligases, followed by incubation with vOTU (0.86 μM; 0.02 ng/ul), AnkOTU (1 μM) or OTUB1* (2 μM). These reactions were then detected with mouse anti-ubiquitin (Enzo Lifesciences; P4D1), or with Anti-Ub^{K48} antibody [1001C] (Abcam, Ab190061). The concentration of vOTU which we identified in B as having no effect on Ub^{K29only} linkages, marginally reduces ubiquitin smears produced by HECTD1 (Lane 4 vs. 3) or UBE3C (Lane 10 vs. 9), although much of the K48 signal is still in the higher polyubiquitinated species. Note that the labelling of the lanes for the Anti-Ub^{K48} antibody panel is accurate. UbiCREST assays using vOTU did not provide additional insight with regards to the architecture of these K29/K48 chains.

Supplementary Fig 7

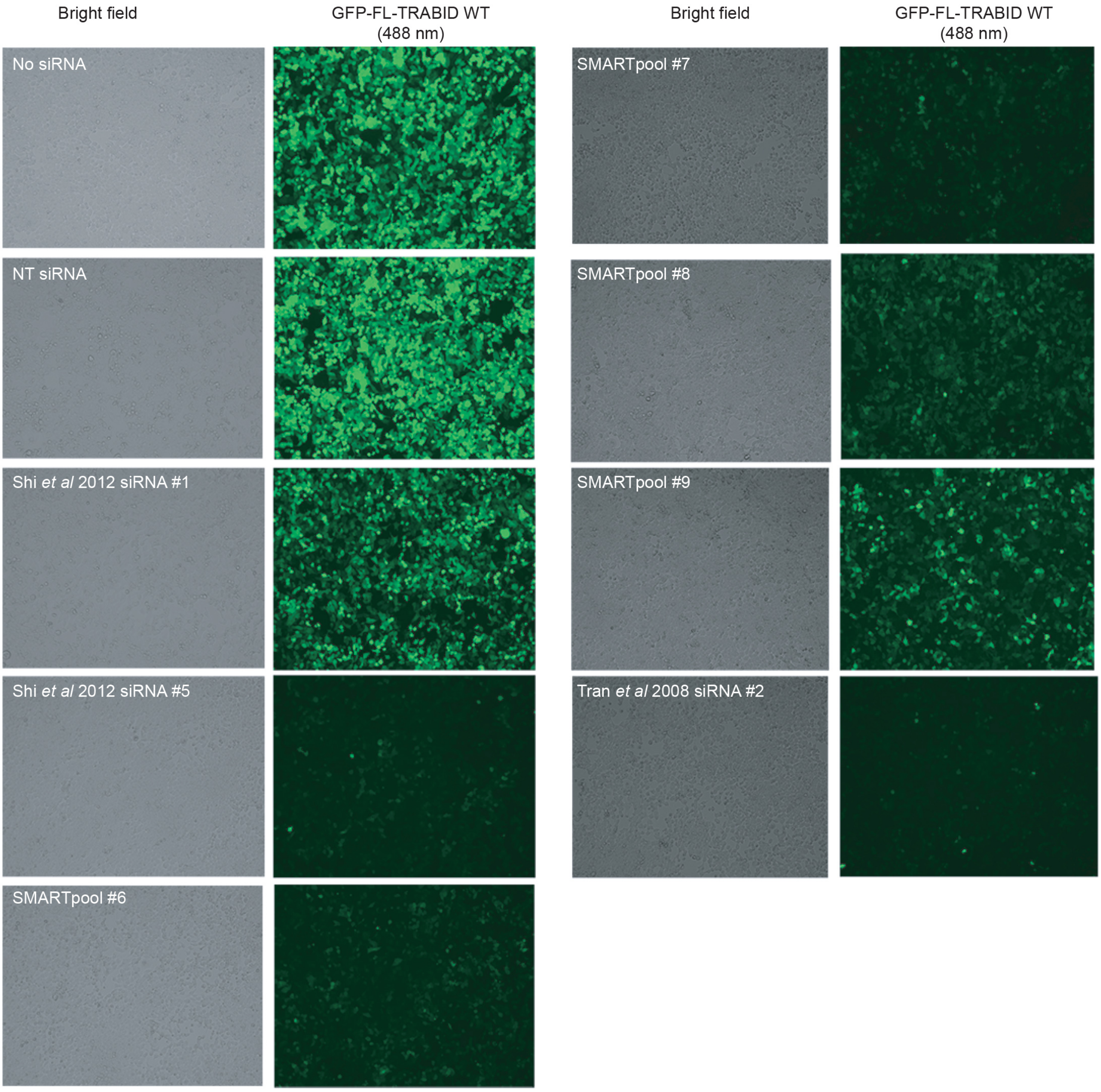

Supplementary Figure 7. Validation of TRABID siRNA in HEK293ET cells.

Given the lack of reliable and validated TRABID antibodies, the efficacy of TRABID siRNA was tested in HEK293ET cells overexpressing pEGFP-FL-TRABID^{WT}. Briefly, HEK293ET were adhered onto Poly-L lysine-coated coverslips, and 200 ng of pEGFP-FL-TRABID plus 20 pmol of siRNA per well/24-well plate were transfected using lipofectamine 2000. Forty-eight hours following

transfection, cells were fixed with 4% paraformaldehyde and imaged on an EVOS Cell Imaging System (ThermoFisher Scientific).

Supplementary Table 1

Sheet 1: Unique peptide count (n= 2,250 proteins)

Sheet 2: Percent coverage (n= 2,250 proteins)

Sheet 3: Unique peptide count & percent coverage (n= 2,250 proteins)

Sheet 4: Initial working list (n= 208 proteins)

Sheet 5: Final working list (n= 215 proteins)

Sheet 6: TRABID OTU-only list (n= 103 proteins)

Sheet 7: TRABID ∆OTU-only list (n= 23 proteins)

Sheet 8: Candidate substrates (n= 50 proteins)

Supplementary Table 2

Absolute quantitation (AQUA) of ubiquitin linkages using heavy/light peptide standards.

References

1. Tran, H., Hamada, F., Schwarz-Romond, T., and Bienz, M. (2008) Trabid, a new positive regulator of Wnt-induced transcription with preference for binding and cleaving K63-linked ubiquitin chains. *Genes & Development* **22**, 528-542
2. Licchesi, J. D. F., Mieszczanek, J., Mevissen, T. E. T., Rutherford, T. J., Akutsu, M., Virdee, S., El Oualid, F., Chin, J. W., Ovaa, H., Bienz, M., and Komander, D. (2011) An ankyrin-repeat ubiquitin-binding domain determines TRABID's specificity for atypical ubiquitin chains. *Nature Structural & Molecular Biology* **19**, 62-71
3. Michel, M. A., Elliott, P. R., Swatek, K. N., Simicek, M., Pruneda, J. N., Wagstaff, J. L., Freund, S. M. V., and Komander, D. (2015) Assembly and specific recognition of k29- and k33-linked polyubiquitin. *Molecular Cell* **58**, 95-109
4. Lam, Y. A., Xu, W., DeMartino, G. N., and Cohen, R. E. (1997) Editing of ubiquitin conjugates by an isopeptidase in the 26S proteasome. *Nature* **385**, 737-740
5. McGouran, J. F., Gaertner, S. R., Altun, M., Kramer, H. B., and Kessler, B. M. (2013) Deubiquitinating enzyme specificity for ubiquitin chain topology profiled by di-ubiquitin activity probes. *Chemistry & Biology* **20**, 1447-1455
6. Hu, M., Li, P., Song, L., Jeffrey, P. D., Chenova, T. A., Wilkinson, K. D., Cohen, R. E., and Shi, Y. (2005) Structure and mechanisms of the proteasome-associated deubiquitinating enzyme USP14. *The EMBO Journal* **24**, 3747-3756
7. Mansour, W., Nakasone, M. A., von Delbrück, M., Yu, Z., Krutauz, D., Reis, N., Kleifeld, O., Sommer, T., Fushman, D., and Glickman, M. H. (2015) Disassembly of Lys11 and mixed linkage polyubiquitin conjugates provides insights into function of proteasomal deubiquitinases Rpn11 and Ubp6. *The Journal of biological chemistry* **290**, 4688-4704
8. You, J., and Pickart, C. M. (2001) A HECT domain E3 enzyme assembles novel polyubiquitin chains. *The Journal of biological chemistry* **276**, 19871-19878
9. Kristariyanto, Y. A., Abdul Rehman, S. A., Campbell, D. G., Morrice, N. A., Johnson, C., Toth, R., and Kulathu, Y. (2015) K29-selective ubiquitin binding domain reveals structural basis of specificity and heterotypic nature of k29 polyubiquitin. *Molecular Cell* **58**, 83-94
10. Ritorto, M. S., Ewan, R., Perez-Oliva, A. B., Knebel, A., Buhrlage, S. J., Wightman, M., Kelly, S. M., Wood, N. T., Virdee, S., Gray, N. S., Morrice, N. A., Alessi, D. R., and Trost, M. (2014) Screening of DUB activity and specificity by MALDI-TOF mass spectrometry. *Nature communications* **5**, 4763
